# Transcriptomic profiling and genetic analyses reveal novel key regulators of cellulase and xylanase gene expression in *Penicillium oxalicum*

**DOI:** 10.1186/s13068-017-0966-y

**Published:** 2017-11-22

**Authors:** Yu-Si Yan, Shuai Zhao, Lu-Sheng Liao, Qi-Peng He, Ya-Ru Xiong, Long Wang, Cheng-Xi Li, Jia-Xun Feng

**Affiliations:** State Key Laboratory for Conservation and Utilization of Subtropical Agro-bioresources, College of Life Science and Technology, Guangxi University, 100 Daxue Road, Nanning, 530004 Guangxi People’s Republic of China

**Keywords:** *Penicillium oxalicum*, Transcriptomic profiling, Transcription factor, Cellulase, Xylanase, Regulation

## Abstract

**Background:**

The transition to a more environmentally friendly economy has prompted studies of modern biorefineries, including the utilization of low-value lignocellulose. The major challenge facing the widespread application of biorefineries is the high cost of enzymes that can efficiently hydrolyze recalcitrant cellulose to sugars. *Penicillium oxalicum* produces large amounts of plant-cell-wall-degrading enzymes, but their production is tightly controlled by complex regulatory networks, resulting in low yields of the native enzymes. Regulatory genes have been the targets of genetic engineering to improve enzyme production in microorganisms. In this study, we used transcriptomic profiling and genetic analyses to screen for and identify novel key regulators of cellulase and xylanase gene expression in *P. oxalicum*.

**Results:**

A comparative analysis of the transcriptomes of *P*. *oxalicum* HP7-1 on different carbon sources, including glucose, wheat bran, and wheat bran plus Avicel, identified 40 candidate genes regulating the expression of cellulolytic enzyme genes. Deletion mutants of 31 candidate genes were constructed in *P*. *oxalicum* ∆*PoxKu70* and 11 resultant mutants showed significant changes in their filter-paper cellulase production compared with the parental strain ∆*PoxKu70*. Among these 11 mutants, Δ*PoxCxrA*, Δ*PoxCxrB*, and Δ*PoxNsdD* showed the most significant reduction in the enzyme production (96.8, 75.9, and 58.5%, respectively). Ten of these 11 genes are here reported to be involved in cellulase production for the first time. Further tests revealed that Δ*PoxCxrA*, Δ*PoxCxrB*, and Δ*PoxNsdD* displayed significantly reduced xylanase production, whereas Δ*PoxCxrA* produced negligible xylanase. Interestingly, Δ*PoxCxrB* and Δ*PoxNsdD* showed significantly increased β-glucosidase production. Real-time quantitative reverse transcription–PCR and an electrophoretic mobility shift assay (EMSA) showed that *PoxCxrA*, *PoxCxrB*, and *PoxNsdD* regulate the expression of one another, but the mode of regulation changes dynamically during the growth of fungal cells in the presence of cellulose. EMSA showed that PoxCxrA, PoxCxrB, and PoxNsdD directly bind the putative promoters of major cellulase and xylanase genes.

**Conclusions:**

We have detected and identified three key new regulatory genes, *PoxCxrA*, *PoxCxrB*, and *PoxNsdD*, that directly and indirectly regulate the expression of cellulase and xylanase genes in *P*. *oxalicum*. This study provides novel insights into the regulatory mechanisms of fungal cellulase and xylanase gene expression.

**Electronic supplementary material:**

The online version of this article (10.1186/s13068-017-0966-y) contains supplementary material, which is available to authorized users.

## Background

Plant cell walls, which consist primarily of cellulose, hemicellulose, and lignin, are the most abundant renewable bioresource on earth. Filamentous fungi, particularly *Penicillium*, *Trichoderma*, *Aspergillus*, and *Neurospora* species, produce a diverse array of enzymes in response to different ecological niches. These include plant-cell-wall-degrading enzymes (CWDEs) that depolymerize the main structural polysaccharide components of plant cell walls into single sugars (glucose or xylose), which can be further converted into liquid fuels and/or other useful chemicals. However, low CWDE production has seriously retarded the industrialization of lignocellulosic biorefineries [1].

*Penicillium* has already received serious attention as an alternative to *Trichoderma reesei* for the production of industrial cellulases for use in lignocellulose saccharification. It has two major advantages over *T*. *reesei*: high β-glucosidase (BGL) activity in extracellular cellulase systems and its superior hydrolytic performance, which is dependent on the high specific activity of cellobiohydrolase 1 (CBH1) [2]. In the genus *Penicillium*, *P. oxalicum* HP7-1, isolated from a decayed subtropical forest soil system in China, produces high cellulase activity for alkaline-pretreated sugarcane bagasse [3, 4], which is the major waste product in the Chinese sugar industry. The whole genomic sequence of *P*. *oxalicum* HP7-1 and its annotation were recently completed, and showed that it has an integrative lignocellulolytic enzyme system for the degradation of plant cell walls, with diverse components [5].

The complexity of the signaling cascades involved and our incomplete knowledge of the relevant transcriptional regulatory networks have hindered the improvement of native CWDE production in filamentous fungi [6]. The CWDE genes are coordinately but differentially regulated in their hosts. The further characterization and manipulation of regulatory networks of CWDE gene expression should allow CWDE yields to be improved via the rational genetic engineering of filamentous fungi.

The transcriptional regulation of cellulolytic gene expression is vital for cellulase biosynthesis and secretion in filamentous fungi, and depends on a regulatory network of multiple positively and negatively acting transcription factors (TFs). This network controls cellulolytic and xylanolytic gene expression in response to multiple external inducers/repressors, such as low-molecular-weight monosaccharides or disaccharides produced by the degradation of polysaccharides, light signaling, and pH [7].

XlnR is the first-identified TF involved in xylan and cellulose degradation, and regulates the expression of xylanolytic and cellulolytic genes induced by d-xylose or cellobiose in *Aspergillus* [8, 9]. Several TFs have subsequently been identified as involved in the degradation of plant biomass. These predominantly include transcription activators, such as CLR-1/ClrA, CLR-2/ClrB [5, 10, 11], VIB1/XprG [12, 13], and Ace3 [14], and carbon catabolite repressors, such as CreA/CRE1/CRE-1 [15–17]. The TF Ace3 is a master regulator of cellulase gene expression and a modulator of xylanase gene expression, which is mediated partly by the XlnR homologue Xyr1 in *T. reesei* [14]. In *N. crassa*, CLR-2 functions as a primary transcriptional activator, directly regulating the transcription of cellulolytic enzyme genes. The transcriptional regulation of *clr*-*2* is controlled by activated CLR-1, and CLR-1 and CLR-2 together constitute the full responses to cellulase gene expression [10, 15]. Another TF, VIB1, indirectly activates the CLR-1/CLR-2-triggered expression of cellulolytic enzyme genes by repressing both Cre1-mediated carbon catabolite repression (CCR) and COL-26/BglR-mediated glucose sensing and metabolism in the early phase of cellulolytic induction [12]. CCR is a global regulatory mechanism that ensures microorganisms, including filamentous fungi, preferentially utilize glucose or other rapidly metabolizable carbon sources over less-favorable carbohydrates. In filamentous fungi that can degrade lignocellulose, such as *Aspergillus*, *Trichoderma*, *Penicillium*, and *Neurospora*, the C2H2-type TF CreA/CRE1/CRE-1 functions as the main repressor in CCR. It directly or indirectly represses the expression of almost all CWDE genes and their TFs involved in the degradation of plant biomass in the presence of glucose [2, 15–17]. In *A. nidulans*, the CreA function depends in part on de novo protein synthesis, and *CreA* expression is not only autoregulated, but is also regulated by CreB–CreC-mediated ubiquitination [18]. COL-26 plays an important role in regulating glucose metabolism in *N*. *crassa*, and functions synergistically with CRE-1 to regulate cellulase gene expression [12].

TFs and their homologues in different fungi also play diverse roles in the regulation of CWDE gene expression, conferring specificity and diversity on the mechanisms regulating the expression of these genes. For example, Xyr1/XlnR activates the transcription of the cellulase and xylanase genes in *T*. *reesei*, *A*. *niger*, and *P*. *oxalicum*, whereas Xlr-1/XlnR only regulates the expression of xylanase genes in *N*. *crassa* and *A*. *nidulans* [15]. In *N*. *crassa*, CLR-1 promotes the transcription of a variety of genes encoding cellulases, hemicellulases, cellodextrin transporters, and proteins involved in protein secretion, whereas its homologue ClrA is less involved in the regulation of cellulase and hemicellulase gene expression in *A*. *nidulans* [7, 10].

In *P*. *oxalicum*, many TFs regulating the expression of cellulase and hemicellulase genes have been identified, including POX02484, POX08522, Ace1, AmyR, Bgl2, CreA, ClrB, ClrB-2, ClrC, and XlnR [5, 17]. Of these, ClrB, CreA, XlnR, and AmyR are important dose-dependent TFs in cellulase production. During the induction of cellulose or cellodextrins, CreA and AmyR repress the expression of cellulase genes, whereas ClrB and XlnR activate their expression. In the early stage of induction, ClrB represses *amyR* expression, which is activated by CreA. AmyR also plays a negative role in the regulation of *clrB* and *xlnR* expression. The transcriptional expression of *amyR*, *clrB*, and *xlnR* is also repressed by CreA in the presence of glucose [17].

The combined manipulation of TFs and their target genes recently enhanced the production of cellulolytic enzymes in *P*. *oxalicum*. The simultaneous deletion of the TF-encoding genes *creA* and *bgl2* and the overexpression of *clrB* improved the filter-paper cellulase (FPase) activity and its extracellular protein concentration over 20- and 10-fold, respectively [19]. When the gene encoding the TF XlnR^A871V^, *clrB*, and two major cellulase genes, *cbh1* and *eg1*, were overexpressed and the carbon catabolite repressor gene *creA* was deleted, the engineered *P*. *oxalicum* strain showed approximately tenfold higher cellulase activity than the wild type [20]. These data suggest that genetically modifying TFs by overexpressing or deleting their genes can efficiently improve the production of cellulolytic enzymes. However, these improvements cannot meet the industrial demand for lignocellulosic biorefineries. Therefore, more-specific regulators of cellulase and xylanase gene expression in *P*. *oxalicum* are still required. These should also will extend our understanding of the mechanisms regulating cellulase and xylanase gene expression and how *P*. *oxalicum* can be engineered to enhance its cellulase and xylanase production.

In this study, the transcriptomes of *P*. *oxalicum* HP7-1 when cultured on different carbon sources were profiled to identify candidate regulators of its cellulolytic and xylanolytic genes. Three novel key regulatory genes that simultaneously regulate the expression of major cellulase and xylanase genes were detected and identified. The three novel regulatory genes were also found to regulate the expression of one another.

## Results

### Forty candidate regulators of cellulolytic gene expression were detected in *P*. *oxalicum* with transcriptomic profiling

To screen for candidate regulators of cellulolytic gene expression in *P. oxalicum*, deep RNA sequencing (RNA-seq) was used to analyze the transcript profiles of *P*. *oxalicum* HP7-1. After medium containing glucose (Glu), wheat bran (WB), or wheat bran plus Avicel (WA) as the sole carbon source was directly inoculated with *P*. *oxalicum* HP7-1, the fungal cells showed significant difference in their cellulase and xylanase activities. In the presence of Glu, *P*. *oxalicum* HP7-1 underwent carbon catabolite repression, and its cellulase and xylanase activities were barely detectable. In the 3 days after inoculation, all the enzymatic activities of strain HP7-1, including those of FPase, endo-glucanase (CMCase), *p*-nitrophenyl-β-cellobiosidase (pNPCase), *p*-nitrophenyl-β-glucopyranosidase (pNPGase), and xylanase, were similar on WB and WA. After day 3 post-inoculation, the FPase, CMCase, pNPCase, and xylanase activities on WA increased sharply compared with those on WB. The maximum FPase activities of strain HP7-1 were 1.45 ± 0.01 and 0.5 ± 0.04 U/mL on WA and WB, respectively, on day 6 after inoculation. Interestingly, the pNPGase activity of HP7-1 on WA was lower than that on WB between days 3 and 5 after inoculation, whereas after day 5, it was higher on WA than on WB (Additional file 1: Figure S1). *P. oxalicum* HP7-1 also produced FPase activity of 0.24 ± 0.07 U/mL on Avicel on day 6 after inoculation.

Glucose is known to induce CCR. WB is a low-cost agricultural waste product, composed predominantly of starch (~ 19%), nonstarch polysaccharides (~ 58%), and crude protein (~ 18%). Among the nonstarch polysaccharides, soluble cello-oligosaccharides are the most significant factors in cellulase production in *P*. *oxalicum* [21]. Although WB and Avicel both induce cellulase production, *P*. *oxalicum* HP7-1 produced more cellulase when grown on WA than when grown on WB or Avicel alone. The cellulase and xylanase activities of *P*. *oxalicum* HP7-1 differed significantly when it was cultured on inducing or repressing carbon sources. Therefore, the transcriptomes of HP7-1 grown on WA, WB, and Glu were screened for candidate regulators of cellulase and xylanase gene expression.

The total RNAs were extracted from the mycelia of *P*. *oxalicum* HP7-1 grown on WA, WB, or Glu for 72 h, and then sequenced. In total, approximately 26–27 million clean reads, 90 bp in length (Accession Number SRA505232), were generated from each sample, constituting an average 80-fold coverage of the *P*. *oxalicum* HP7-1 genome [5]. The average clean reads were mapped to 8623 predicted protein-coding genes in the HP7-1 genome (Additional file 2: Table S1). The Pearson’s correlation coefficients were high (*R* ≥ 0.80) for the three biological replicates produced under each set of culture conditions (Additional file 3: Figure S2).

**Fig. 1:**
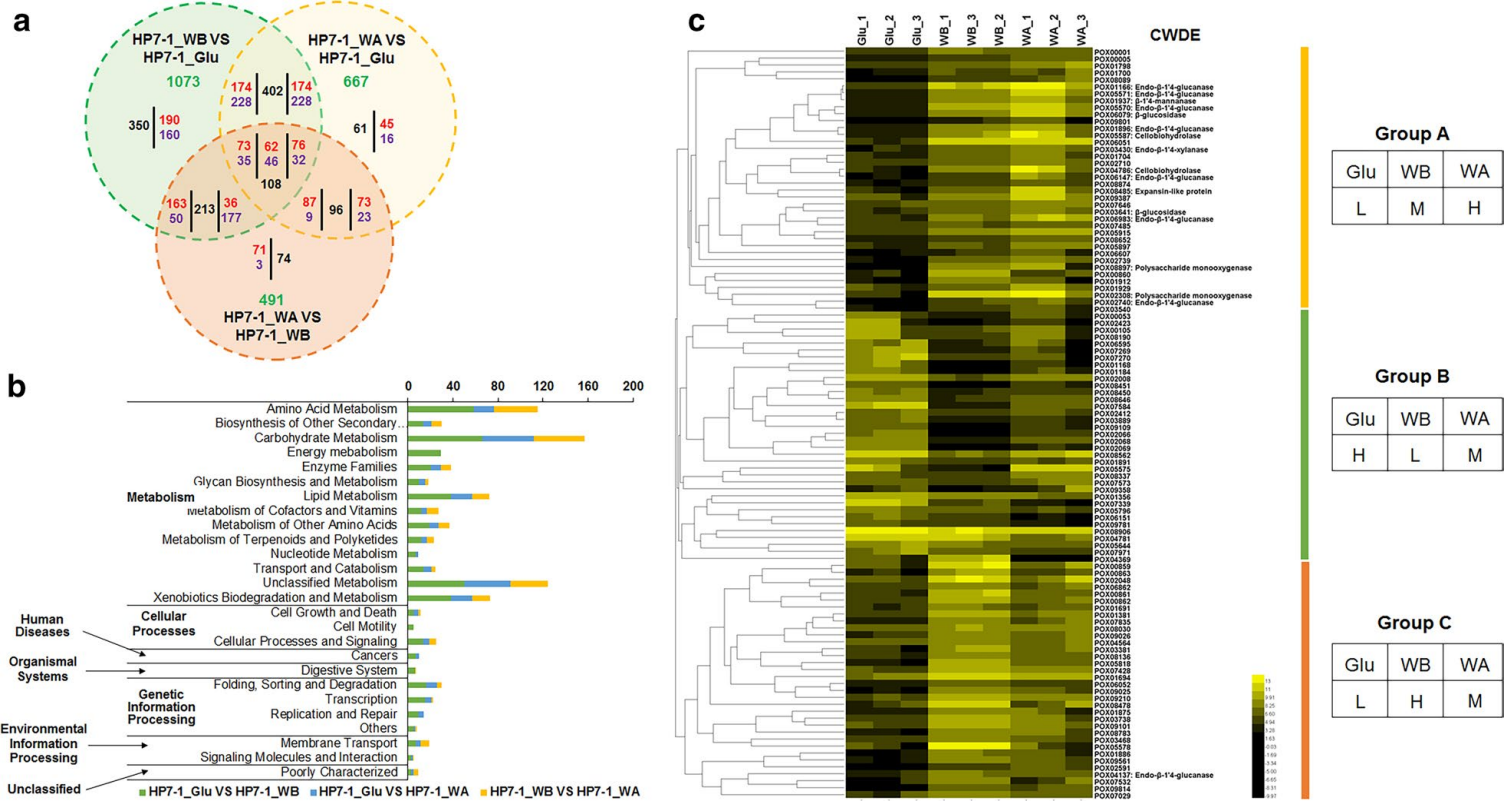
Comparative analysis of the transcriptomes of *P. oxalicum* HP7-1 cultured in medium containing glucose (Glu), wheat bran (WB), or wheat bran plus Avicel (WA) as the carbon source. **a** Venn diagram showing the numbers of unique and shared differentially expressed genes from different transcriptomic comparisons. Red numbers represent upregulated genes; purple numbers represent downregulated genes; black numbers are the total upregulated and downregulated genes; green numbers represent the total numbers of differentially expressed genes. The numbers in overlapping areas show the results of different comparative transcriptomes. In the overlapping areas shared by two compared transcriptomes, numbers on the left refer to the comparison on the left or above the overlapping area, and numbers on the right represent the comparison below the overlapping area. In the overlapping area shared by three comparative transcriptomes, the left column, middle column, and right column represent HP7-1_WB vs HP7-1_Glu, HP7-1_WA vs HP7-1_WB, and HP7-1_WA vs HP7-1_Glu, respectively. **b** Kyoto encyclopedia of genes and genomes (KEGG) annotation of the proteins encoded by genes with significantly altered transcription. The screening criteria for altered genes were |log_2_ fold change| ≥ 0.8 and probability ≥ 0.8. **c** Heatmap showing the genes identified as differentially expressed on three carbon sources. *L* low expression in Glu culture, *M* moderate expression in WB culture, *H* high expression in WA culture. *CWDE* plant-cell-wall-degrading enzyme

The clean reads obtained were then mapped onto the genome of *P*. *oxalicum* HP7-1 with the software BWA [22] and Bowtie [23]. The expression levels (fragments per kilobase of exon per million mapped reads, FPKM) of the genes were calculated with the software package RSEM [24], and the differentially expressed genes were detected with NOISeq in the R package [25]. Overall, a comparative analysis of these genes identified 1073, 667, and 491 genes as significantly differentially expressed between HP7-1_WB and HP7-1_Glu, HP7-1_WA and HP7-1_Glu, and HP7-1_WA and HP7-1_WB, respectively (|log_2_ fold change| ≥ 0.8 and probability ≥ 0.8 were used as the thresholds). These gene sets contained 600, 382, and 242 upregulated genes, respectively, which mainly functioned in metabolic pathways, such as carbohydrate metabolism and energy metabolism (Fig. 1a, b). Among these differentially expressed genes, 108 were co-expressed on all the carbon sources tested (Additional file 4: Table S2). They formed three major groups with internally similar expression patterns in a heatmap hierarchical clustering assay: Group A containing 37 genes, Group B containing 37 genes, and Group C containing 34 genes (Fig. 1c).

Group A consisted of 37 genes displaying low expression (L) in Glu culture, moderate expression (M) in WB culture, and high expression (H) in WA culture. Of these, 11 were cellulase genes, including two cellobiohydrolase genes (*cbhs*; *POX04786*/*Cel6A* and *POX05587*/*Cel7A*-*2*), seven endo-β-1,4-glucanase genes (*egs*; *POX01166*/*Cel5B*, *POX01896*/*Cel5C*, *POX02740*, *POX05570*/*Cel45A*, *POX05571*/*Cel7B*, *POX06147*/*Cel5A*, and *POX06983*), two β-glucosidase genes (*bgls*; *POX03641* and *POX06079*). Two hemicellulase genes (*POX01937* and *POX03430*), three genes encoding enzymes with predicted activity against polysaccharides (*POX02308*, *POX08485*, and *POX08897*), and seven genes encoding major facilitator superfamily (MFS) members, including cellodextrin transporters (POX06051/CdtC and POX05915/CdtD [26]) were included (Fig. 1c). These data strongly support the finding that the strongest FPase activity (1.45 U/mL) in *P*. *oxalicum* HP7-1 was observed in WA culture (Additional file 1: Figure S1).

**Fig. 2:**
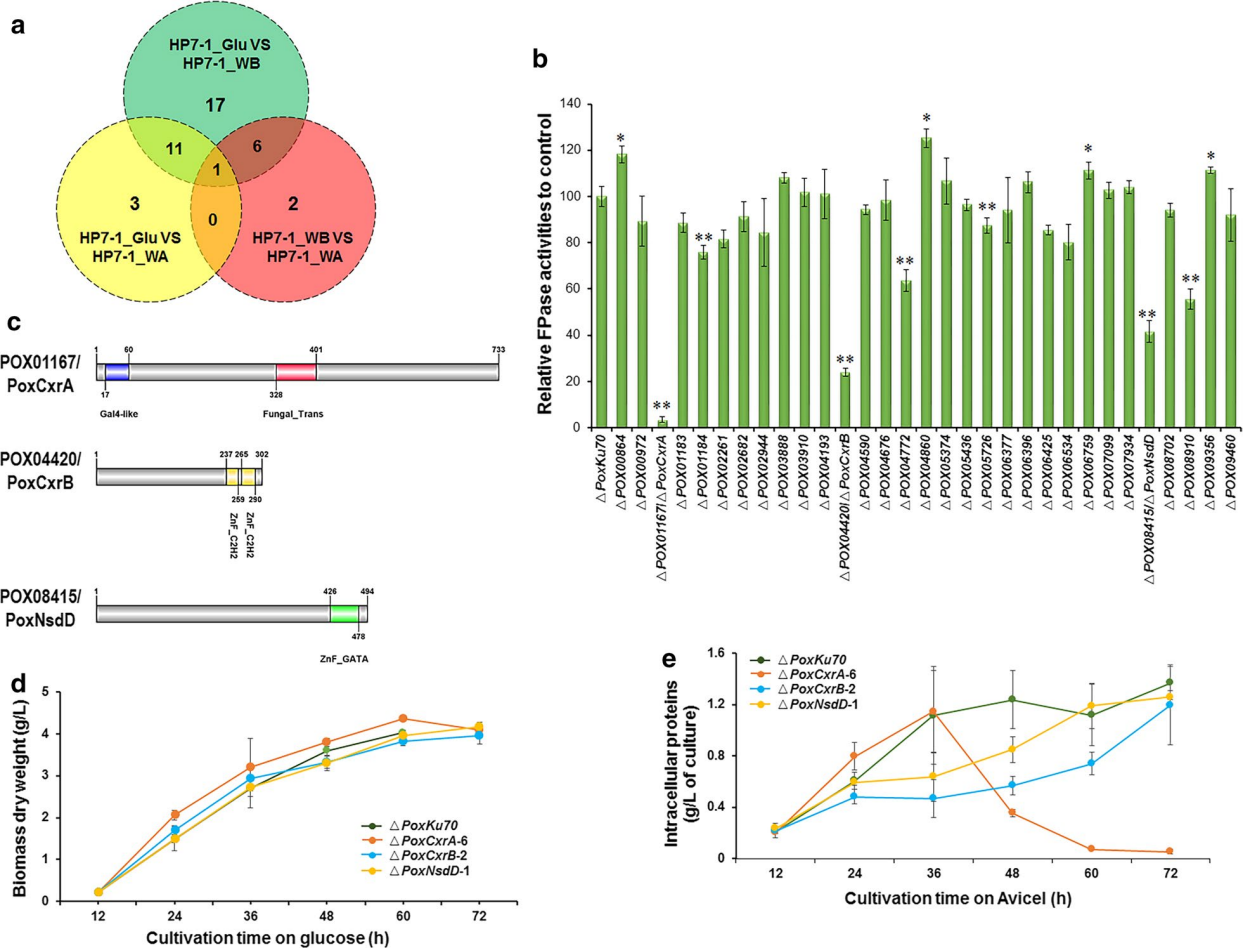
Screening for and identification of novel regulators required for cellulase and xylanase production in *P. oxalicum*. **a** Venn diagram showing unique and shared candidate regulators selected on different carbon sources. The filtering criteria were |log_2_ fold change| ≤ 1.5, *P* ≤ 0.01, and probability ≥ 0.8. HP7-1_Glu, the HP7-1 strain grown in glucose medium; HP7-1_WB, the HP7-1 strain grown in wheat bran medium; HP7-1_WA, the HP7-1 strain grown in wheat bran plus Avicel medium. **b** Filter-paper cellulase activities of deletion mutants of the candidate regulatory genes on Avicel as the sole carbon source 6 days after inoculation. *P ≤ 0.05 and **P ≤ 0.01 between the deletion mutants and the parental strain Δ*PoxKu70*, assessed with Student’s *t* test. **c** Modular architecture of POX01167/PoxCxrA, POX04420/PoxCxrB, and POX08415/PoxNsdD. (D and E) Growth profiles of Δ*PoxCxrA*-6, Δ*PoxCxrB*-2, and Δ*PoxNsdD*-1 on glucose and Avicel, respectively. Data are the means of three biological replicates

By screening the genome of *P*. *oxalicum* HP7-1, we detected 484 genes encoding predicted TFs [5]. Of these, 35, 15, and 9 encoded putative TFs and were differentially expressed in comparisons of HP7-1_WB and HP7-1_Glu, HP7-1_WA and HP7-1_Glu, and HP7-1_WA and HP7-1_WB, respectively, when |log_2_ fold change| ≥ 1.5 and probability ≥ 0.8 were used as the thresholds. Of these, only *POX01184* was differently expressed in all three comparisons. Eleven genes were shared in both comparisons HP7-1_WB vs HP7-1_Glu and HP7-1_WA vs HP7-1_Glu, and six were shared in both HP7-1_WB vs HP7-1_Glu and HP7-1_WA vs HP7-1_WB (Fig. 2a). In total, 40 candidate genes encoding putative TFs were detected, which appeared at least once in all the comparisons of the transcriptomes expressed on the different carbon sources (WA, WB, and Glu). The expression of these genes was induced or repressed to varying degrees by the different carbon sources, ranging from 1.5 < |log_2_ fold change| < 6.25 (Additional file 5: Table S3).

The proteins encoded by the candidate genes were classified into eight types, and about 55% of them contained a zinc finger structure and 15% were homeodomain-like proteins. Seven candidates were homologues of known regulators identified in cellulolytic fungi, including six proteins that regulate cellulase and/or xylanase gene expression (POX01960/ClrB [PoxClrB] [5], POX00972/ClrC [PoxClrC] [17], POX04860/PDE_07199 [17], POX06534/BrlA [PoxBrlA] [27], POX08522 [5], and POX02768/PacC [PoxPacC] [28]), and one protein (POX07099/FlbD [PoxFlbD] [29]) that regulates the expression of genes associated with fungal conidiation (Additional file 5: Table S3).

### Ten novel regulators are required for cellulase production in *P*. *oxalicum*

We attempted to knock-out the 40 candidate genes in the mutant Δ*PoxKu70* [5], which allows high-frequency gene deletion through homologous recombination. *PoxClrB* and *POX08522* were previously knocked-out and identified as regulators of cellulase and xylanase genes when *P*. *oxalicum* HP7-1 was grown on Avicel [5]. Ultimately, 31 deletion mutants were constructed and confirmed with a PCR analysis (Additional file 6: Figure S3) using gene-specific primers (Additional file 7: Table S4), so the deletion of the target genes in Δ*PoxKu70* was 82% successful. Eleven mutants showed significantly altered FPase activities, ranging from 11.2 to 96.8% of the activity of the parental strain Δ*PoxKu70* (*P* ≤ 0.05, Student’s *t* test; Fig. 2b). Of the genes deleted in these 11 mutants, 10 encoded regulators found to be involved in cellulase production in the filamentous fungi for the first time in this study: *POX00864*, *POX01167*, *POX01184*, *POX04420*, *POX04772*, *POX05726*, *POX06759*, *POX08910*, *POX08415*, and *POX09356* (Table 1). The other gene, *POX04860*, encoded the protein homologue of PDE_07199 from *P*. *oxalicum* 114-2, which negatively affects cellulase production, as described previously [17]. Importantly, Δ*POX01167* lost almost all its FPase activity when grown on Avicel. Δ*POX04420* and Δ*POX08415* showed 75.9 and 58.5% reduction in FPase activity compared with the parental strain Δ*PoxKu70* when grown on Avicel, respectively (Fig. 2b). These mutants ranked as the top three in the “hierarchy” of FPase activity, and were selected for further study. Δ*POX01167*, Δ*POX04420*, and Δ*POX08415* were also confirmed with a Southern hybridization analysis (Additional file 6: Figure S3) with specific probes (Additional file 7: Table S4) to exclude the possibility that multiple copies of the deletion cassette were inserted into the Δ*PoxKu70* genome.

**Table 1:** Regulatory genes detected in this study that regulate cellulase production in *P. oxalicum* HP7-1

Gene ID	GenBank accession number	InterPro annotation	Domain description	Known homologous TFs	Identity (%)	FPase activity of mutant relative to parent strain (%)
*POX00864*	KY860734	IPR001138	Zinc finger, Zn2Cys6 type	NA	NA	118.3 ± 3.6
*POX01167*	KY368172	IPR001138 IPR007219	Zinc finger, Zn2Cys6 type; Fungal_Trans	NA	NA	3.6 ± 1.3
*POX01184*	KY860735	IPR011991	Winged helix repressor DNA-binding domain	NA	NA	76.0 ± 3.0
*POX04420*	KY368173	IPR007087	Zinc finger, C2H2 type	NA	NA	24.1 ± 1.7
*POX04772*	KY860736	IPR009071	High mobility group box (HMG)	*A. nidulans* FGSC A4 HmbB	41	63.8 ± 4.8
*POX04860*	KY922971	IPR009057	Homeodomain-like	*P*. *oxalicum* 114-2 PDE_07199	99	125.2 ± 4.1
*POX05726*	KY860737	IPR007087	Zinc finger, C2H2 type	NA	NA	87.5 ± 3.3
*POX06759*	KY860738	IPR001138	Zinc finger, Zn2Cys6 type	*Nectria haematococca mpVI* CTF1 beta	34	111.2 ± 3.8
*POX08415*	KY368171	IPR000679	Zinc finger, GATA type	*A. nidulans* FGSC A4 NsdD	57	41.5 ± 4.7
*POX08910*	KY860739	IPR009057 IPR007526	Homeodomain-like; SWIRM domain	NA	NA	55.6 ± 4.5
*POX09356*	KY860740	IPR001138	Zinc finger, Zn2Cys6 type	NA	NA	111.3 ± 1.4

*IPR* InterPro database (http://www.ebi.ac.uk/interpro/scan.html), *TF* transcription factor, *NA* not annotated, *CTF1 beta* cutinase transcription factor 1 beta, *FPase* filter-paper cellulase

### PoxCxrA, PoxCxrB, and PoxNsdD are zinc finger TFs

*POX01167* encodes a polypeptide of 733 amino acids. A BLAST analysis at the National Center for Biotechnology Information (NCBI) showed that POX01167 contains a GAL4-like Zn_2_Cys_6_ binuclear cluster DNA-binding domain (cd00067) and a fungal TF regulatory middle homology region (cd12148) (Fig. 2c). POX01167 is conserved in *P*. *oxalicum* (i.e., 99% identity with PDE_09277 [EPS34263.1] in *P*. *oxalicum* 114-2), and shares weak identity with proteins in other filamentous fungi that are capable of lignocellulosic degradation (i.e., 66% with EN45_038550 [KZN93671.1] in *P. chrysogenum* P2niaD18, and 44% with ANI_1_1528064 [XP_001391255.2] in *A*. *niger* CBS 513.88).

*POX04420* encodes a polypeptide of 302 amino acids, which contains a two-zinc-finger double domain (pfam13456) (Fig. 2c). A protein alignment with BLAST showed that POX04420 is also conserved in *P*. *oxalicum* (i.e., 99% identity with PDE_03425 [EPS28479.1] in *P*. *oxalicum* 114-2), but shares low identity with proteins in other cellulolytic fungi (i.e., 53% with ANI_1_990104 [XP_001395874.1] in *A*. *niger* CBS 513.88].

POX08415 is a 494-amino acid polypeptide, which contains a conserved GATA-type zinc-finger DNA-binding domain with the amino acid sequence C-X_2_-C-X_18_-C-X_2_-C at its C-terminus. A BlastP analyses showed that POX08415 shares 99% identity with PDE_02029 (EPS27088.1) in *P*. *oxalicum* 114-2, and 57–64% identity with NsdD in *A*. *nidulans* FGSC4 (XP_660756.1), *A*. *flavus* NRRL3357 (XP_002376041.1), and *A*. *fumigatus* AF293 (XP_754237.1).

In subsequent analyses, POX01167 and POX04420 were redesignated PoxCxrA (cellulolytic and xylanolytic regulator A in *P. oxalicum*) and PoxCxrB, respectively. POX08415 was renamed PoxNsdD (NsdD in *P. oxalicum*) based on the name of its homolog NsdD in *Aspergillus*.

A neighbor-joining phylogenetic tree indicated that PoxCxrA and PoxCxrB of *P*. *oxalicum* are closely related to the corresponding homologues in other cellulolytic fungi, including *Aspergillus* sp. and *Talaromyces* sp., but not to those in *T. reesei* or *N. crassa*, whereas PoxNsdD is closely related to the homologues in *T. reesei*, *N. crassa*, and *Talaromyces* sp., but not to that in *Aspergillus* sp. (Additional file 8: Figure S4).

### Growth, cellulase and xylanase production of Δ*PoxCxrA*, Δ*PoxCxrB*, and Δ*PoxNsdD* were impaired on Avicel

To determine whether these proteins affect *P*. *oxalicum* cell growth, the growth of Δ*PoxCxrA* (Δ*POX01167*), Δ*PoxCxrB* (Δ*POX04420*), and Δ*PoxNsdD* (Δ*POX08415*) was compared with that of the parental strain Δ*PoxKu70* when they were directly inoculated into medium containing glucose or Avicel as the sole carbon source. All three mutants accumulated similar amounts of mycelial biomass to that accumulated by Δ*PoxKu70* in glucose medium (Fig. 2d), indicating that the knock-out of none of the three genes affected *P*. *oxalicum* growth when it utilized glucose as the carbon source.

In contrast, the mycelial intracellular proteins of Δ*PoxCxrA* were similar to those of Δ*PoxKu70* before culture for 36 h in Avicel medium, but dropped sharply to approximately a negligible level between 36 and 72 h. Δ*PoxNsdD* produced a similar amount of intracellular protein to Δ*PoxKu70* during culture for 24 h, but after that time, the amount began to decrease relative to that produced by Δ*PoxKu70*. Notably, by 60 h, the amount of intracellular protein in Δ*PoxNsdD* was similar to that in Δ*PoxKu70*. Moreover, Δ*PoxCxrB* contained less mycelial intracellular protein than either Δ*PoxKu70* or Δ*PoxNsdD* during the whole culture period (Fig. 2e). These results clearly indicate that *PoxCxrA*, *PoxCxrB*, and *PoxNsdD* are required for fungal growth on Avicel.

**Fig. 3:**
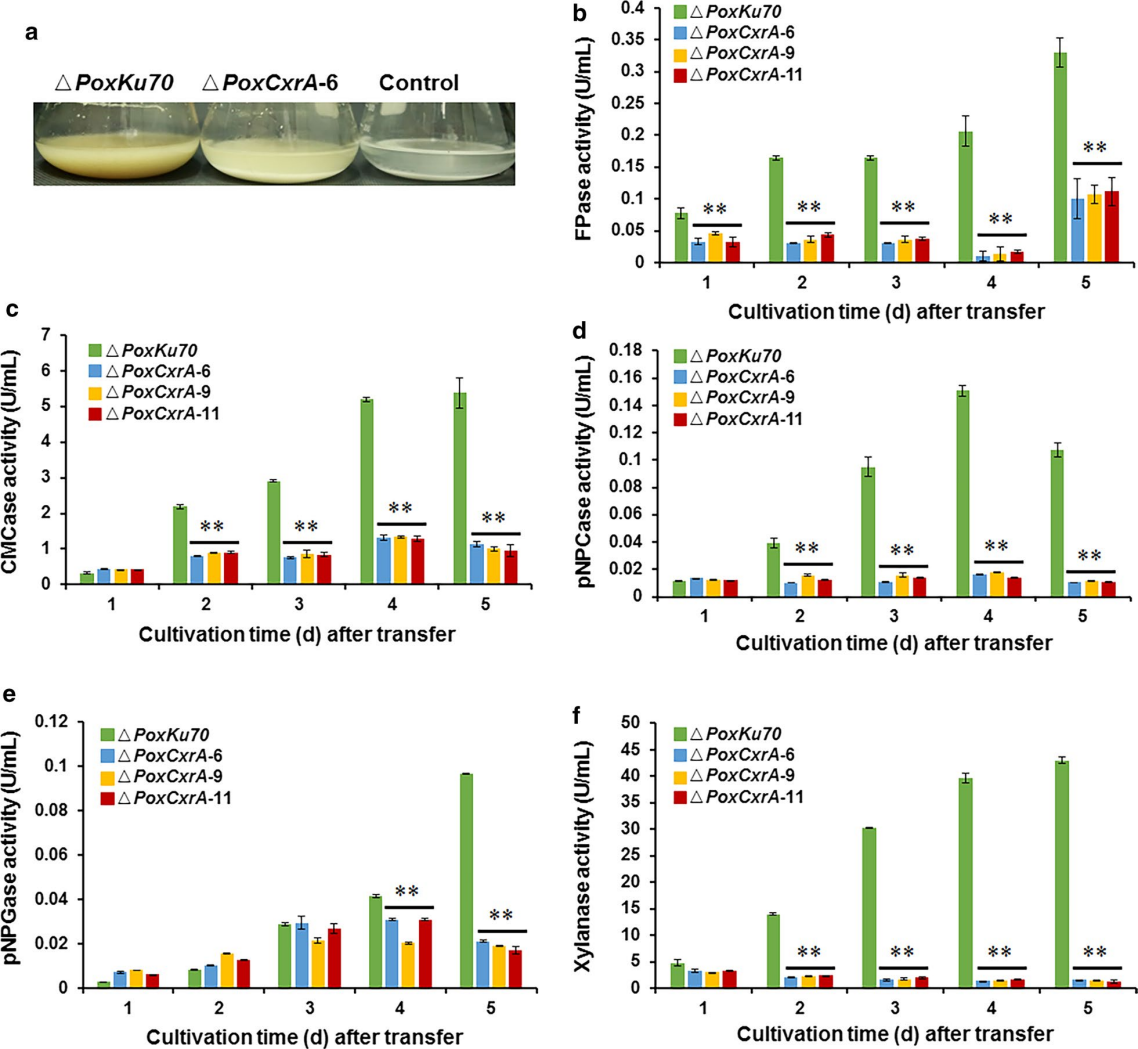
Deletion of *PoxCxrA* abolished the utilization of cellulose by *P. oxalicum*. **a** Growth of Δ*PoxCxrA* and Δ*PoxKu70* on Avicel for 4 days after a shift from glucose; medium without inoculant was used as the blank control. (**b**–**f**) FPase, CMCase, pNPCase, pNPGase, and xylanase activities, respectively, of crude enzymes from Δ*PoxCxrA* and Δ*PoxKu70* after a shift from glucose to Avicel. Crude enzymes were produced by fungal strains cultured on 2% Avicel as the sole carbon source. Enzyme activities were measured 1, 2, 3, 4, and 5 days after the shift. Data are the means of three biological replicates. *FPase* filter-paper cellulase, *CMCase* carboxymethylcellulase, *pNPCase p*-nitrophenyl-β-cellobiosidase, *pNPGase p*-nitrophenyl-β-glucopyranosidase

Based on the growth of Δ*PoxCxrA* after its direct inoculation into Avicel medium (Fig. 2e), we hypothesized that Δ*PoxCxrA* might not respond to complex and recalcitrant carbon sources. To test this hypothesis, we first investigated Δ*PoxCxrA* growth during a shift experiment from glucose to Avicel. The growth of Δ*PoxCxrA* was impaired (Fig. 3a) and the activities of its extracellular cellulase and xylanase were significantly reduced by the shift (Fig. 3b–f).

**Fig. 4:**
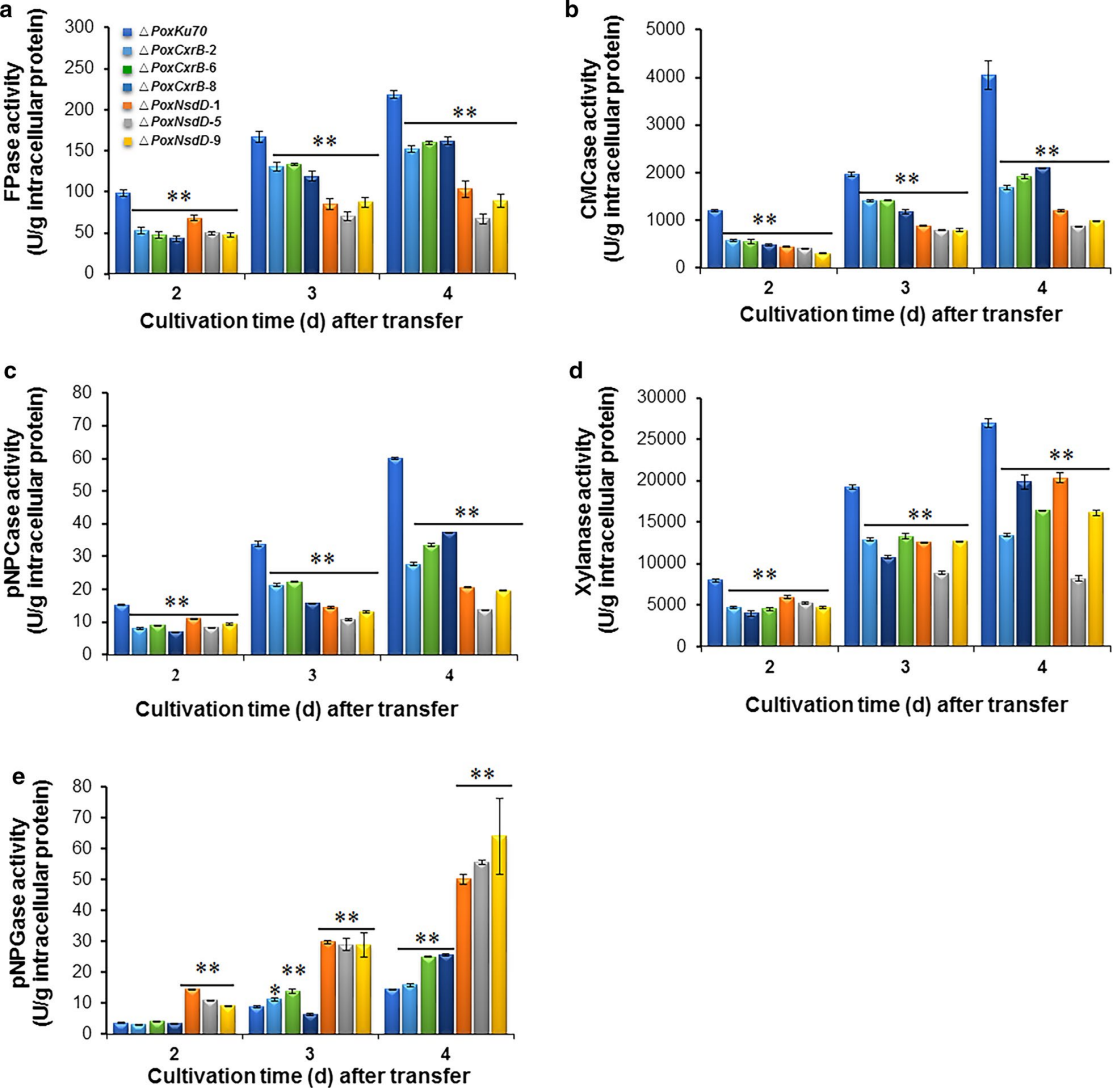
Activities of crude enzymes from Δ*PoxCxrB*, Δ*PoxNsdD*, and Δ*PoxKu70* after a shift from glucose to Avicel. Crude enzymes were produced by fungal strains grown in 2% Avicel as the sole carbon source. Enzymatic activity was measured 2, 3, and 4 days after the shift. *P ≤ 0.05 and **P ≤ 0.01 between the regulatory gene mutants and the parental strain Δ*PoxKu70*, assessed with Student’s *t* test. **a** Filter-paper cellulase (FPase) activity. **b** Endo-glucanase (CMCase) activity. **c** *p*-Nitrophenyl-β-cellobiosidase (pNPCase) activity. **d** Xylanase activity. **e** *p*-Nitrophenyl-β-glucopyranosidase (pNPGase) activity

A shift experiment from glucose to Avicel was used to investigate the cellulase and xylanase production by Δ*PoxCxrB* and Δ*PoxNsdD* in the presence of Avicel. The production of FPase, CMCase, pNPCase, and xylanase by the two deletion mutants was significantly lower than that of Δ*PoxKu70* (*P* ≤ 0.05, Student’s *t* test) (Fig. 4a–d). Interestingly, the pNPGase activity in the mutant Δ*PoxNsdD* increased on average by 217–293%, whereas it only increased by 53.2% in the mutant Δ*PoxCxrB* on day 5 after induction (*P* ≤ 0.01, Student’s *t* test) compared with the activity in Δ*PoxKu70* (Fig. 4e).

**Fig. 5:**
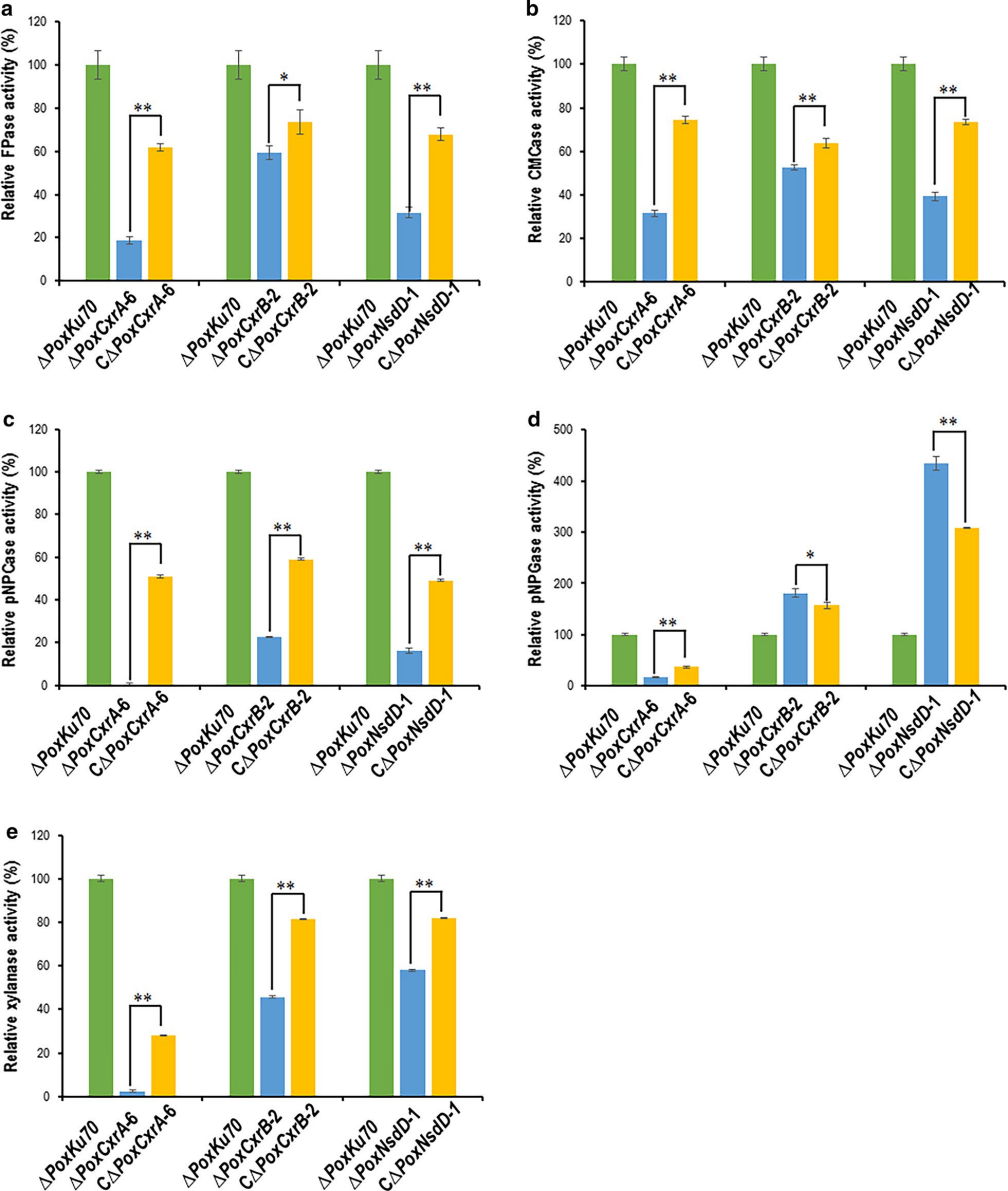
Activities of crude enzymes from the complementary strains CΔ*PoxCxrA*, CΔ*PoxCxrB* and CΔ*PoxNsdD* after a shift from glucose to Avicel. Crude enzymes were produced by fungal strains grown in 2% (w/v) Avicel as the sole carbon source for 4 days after the shift. **P ≤ 0.01 between the complementary strains and the regulatory gene mutants, assessed with Student’s *t* test. *P ≤ 0.05 between CΔ*PoxCxrB* and Δ*PoxCxrB* assessed with Student’s *t* test. **a** Filter-paper cellulase (FPase) activity. **b** Endo-glucanase (CMCase) activity. **c** *p*-Nitrophenyl-β-cellobiosidase (pNPCase) activity. **d** *p*-Nitrophenyl-β-glucopyranosidase (pNPGase) activity. **e** Xylanase activity

To further verify that the changes in cellulase and xylanase production by the mutants Δ*PoxCxrA*, Δ*PoxCxrB* and Δ*PoxNsdD* resulted from the specific deletion of the corresponding gene, the complemented strain of each of the three mutants was constructed, confirmed by PCR (Additional file 7: Table S4 and Additional file 9: Figure S5), and then was tested for enzyme production when grown on Avicel for 4 days after a transfer from glucose to Avicel as described in “Methods” section. The results showed that the complemented strain CΔ*PoxCxrA* could produce 51–74.6% of cellulase activities and 28.2% of xylanase activity of the parent strain ∆*PoxKu70*, which were significantly higher than those of the mutant strain Δ*PoxCxrA* (*P* ≤ 0.01, Student’s *t* test; Fig. 5a–e). Moreover, the complemented strains CΔ*PoxCxrB* and CΔ*PoxNsdD* produced 49.1–73.5% of cellulase activities except for pNPGase activity and ~ 81% of xylanase activity of the ∆*PoxKu70*, which were also significantly higher than those of Δ*PoxCxrB* and Δ*PoxNsdD* (*P* ≤ 0.05, Student’s *t* test; Fig. 5a–c, e). The pNPGase activity of C∆*PoxCxrB* and CΔ*PoxNsdD* was 156.6 and 308.0% of that of ∆*PoxKu70*, respectively, and was significantly lower than that of ∆*PoxCxrB* and Δ*PoxNsdD* (*P* ≤ 0.05, Student’s *t* test; Fig. 5d). These data confirmed that the alterations in cellulase and xylanase production by the mutants Δ*PoxCxrA*, Δ*PoxCxrB* and Δ*PoxNsdD* resulted from the deletion of genes *PoxCxrA*, *PoxCxrB* and *PoxNsdD*.

### Quantitative reverse transcription (RT)-PCR shows that *PoxCxrA*, *PoxCxrB*, and *PoxNsdD* regulate one another and the expression of cellulase and xylanase genes

**Fig. 6:**
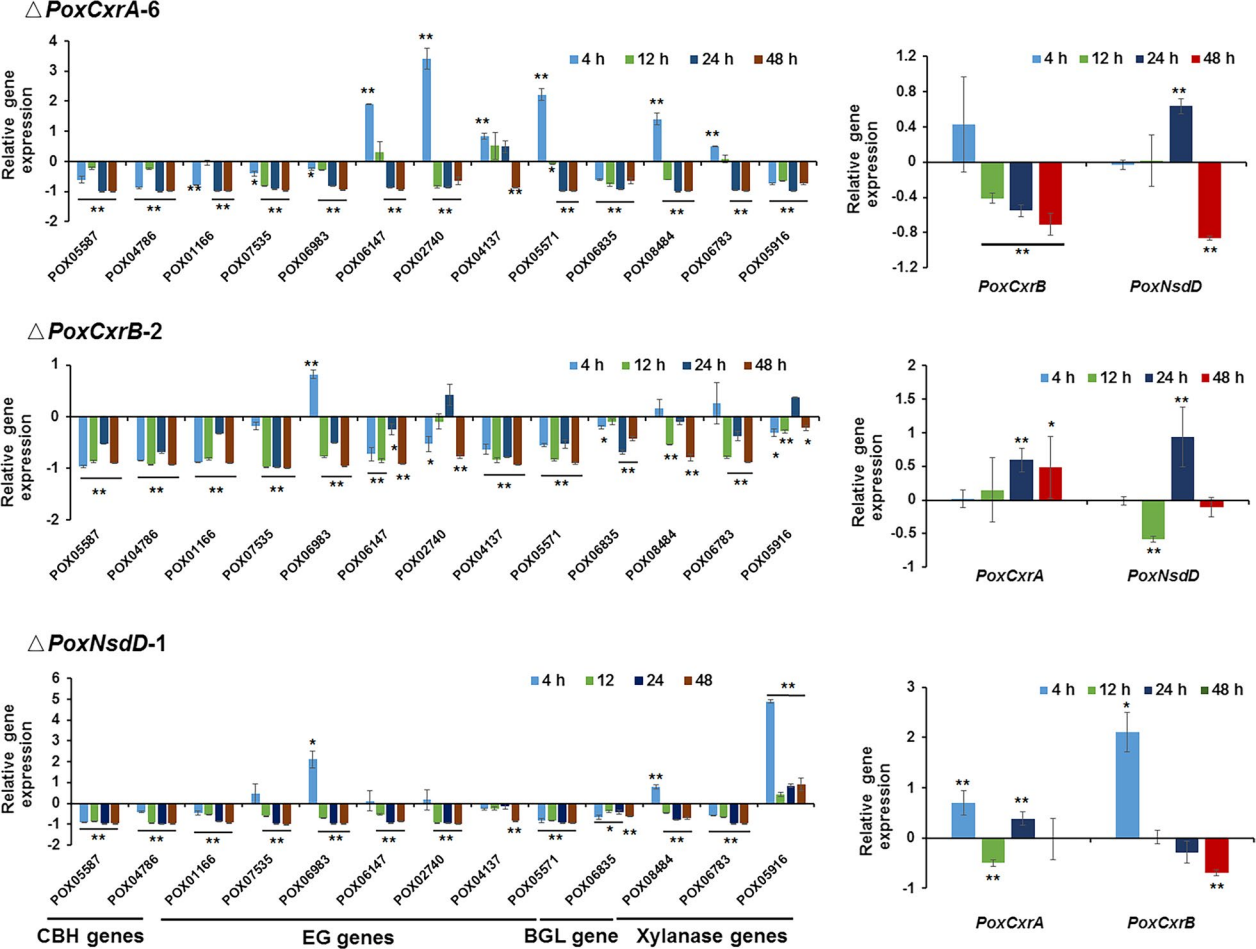
Regulation of gene expression by *PoxCxrA*, *PoxCxrB*, and *PoxNsdD.* Expression levels of cellulase and xylanase genes and the newly identified regulatory genes in the Δ*PoxCxrA*-6, Δ*PoxCxrB*-2, and Δ*PoxNsdD*-1 mutants at four different time points (4, 12, 24, and 48 h) after the shift from glucose to Avicel, determined with RT-qPCR. Relative expression on the y axis is the difference value for the transcription of the genes in the deletion mutants. *P ≤ 0.05 and **P ≤ 0.01 between the samples and Δ*PoxKu70*, assessed with Student’s *t* test. All experiments were performed independently in triplicate at least

To comprehensively analyze the regulatory functions of the identified regulators, mutants Δ*PoxCxrA*, Δ*PoxCxrB*, and Δ*PoxNsdD* were subjected to an RT-qPCR analysis, using as the control Δ*PoxKu70* grown under the same culture conditions. The transcription levels of the major cellulase and xylanase genes and *PoxCxrA*, *PoxCxrB*, and Δ*PoxNsdD* in Δ*PoxCxrA*, Δ*PoxCxrB*, and Δ*PoxNsdD* were measured 4, 12, 24, and 48 h after the shift from glucose to Avicel (Fig. 6). The cellulase and xylanase genes tested included two *cbhs* (*POX05587*/*Cel7A*-*2* and *POX04786*/*Cel6A*), seven *egs* (*POX01166*/*Cel5B*, *POX02740*, *POX04137*, *POX05571*/*Cel7B*, *POX06147*/*Cel5A*, *POX06983*, and *POX07535*/*Cel12A*), one *bgl* (*POX06835*/*Bgl3A*), and three *xyns* (*POX05916*, *POX06783*/*Xyn11A*, and *POX08484*/*Xyn11B*).

In Δ*PoxCxrA*, the transcripts of almost half the cellulase and xylanase genes were significantly downregulated to 26.0–87.8% at 4 h of induction, whereas the remaining genes were upregulated by 50.0–341.4% (*P* ≤ 0.05, Student’s *t* test). After 4 h, the transcription of almost all the genes had decreased by 22.3–99.5% (*P* ≤ 0.05, Student’s *t* test) in Δ*PoxCxrA* compared with that in the parental strain Δ*PoxKu70* (Fig. 6).

In Δ*PoxCxrA*, the transcripts of the *PoxCxrB* gene were downregulated to some extent (by 41.2–70.6%) after 4 h of induction on Avicel (*P* ≤ 0.05, Student’s *t* test). These effects became more severe as the induction time increased. The transcripts of *PoxNsdD* in Δ*PoxCxrA* were upregulated by 63.6% at 24 h, and then downregulated by 86.5% at 48 h (*P* ≤ 0.01, Student’s *t* test) compared with those in the parental strain Δ*PoxKu70* (Fig. 6).

The expression of cellulase and xylanase genes in Δ*PoxCxrB* and Δ*PoxNsdD* was also investigated. Almost all the genes tested were downregulated in Δ*PoxCxrB* and Δ*PoxNsdD* during the whole induction period. Notably, the expression of the *e.g.* gene *POX06983* increased by 82.2 and 212.0% in the Δ*PoxCxrB* and Δ*PoxNsdD* mutants, respectively, after induction for 4 h, and the expression of an important *xyn* gene, *POX08484*/*Xyn11B*, was upregulated by 79.1% in mutant Δ*PoxNsdD* (*P* ≤ 0.05, Student’s *t* test). The expression of another *xyn* gene, *POX05916*, was also negatively regulated by 43.0–489.7% by PoxNsdD during the whole induction period on Avicel (*P* ≤ 0.05, Student’s *t* test; Fig. 6).

When the expression levels of *PoxCxrA* and *PoxNsdD* were tested in the mutant Δ*PoxCxrB*, the transcripts of *PoxCxrA* were 59.2 and 48.8% higher than that in Δ*PoxKu70* after 24 h and 48 h of induction (*P* ≤ 0.05, Student’s *t* test), respectively. However, *PoxNsdD* expression first decreased by 58.2% at 12 h, but subsequently increased by 93.4% at 24 h (*P* ≤ 0.01, Student’s *t* test; Fig. 6).

In Δ*PoxNsdD*, the transcripts of *PoxCxrA* increased by 69.7 and 38.2% after 4 and 24 h of induction, respectively, whereas they decreased by 50% after 12 h. Interestingly, *PoxCxrB* expression in Δ*PoxNsdD* increased by 210.6% after induction for 4 h, whereas after 48 h, it had decreased by 69.3% compared with that in Δ*PoxKu70* (*P* ≤ 0.05, Student’s *t* test; Fig. 6).

### PoxCxrA, PoxCxrB, and PoxNsdD bind to the promoter regions of targeted genes in vitro

**Fig. 7:**
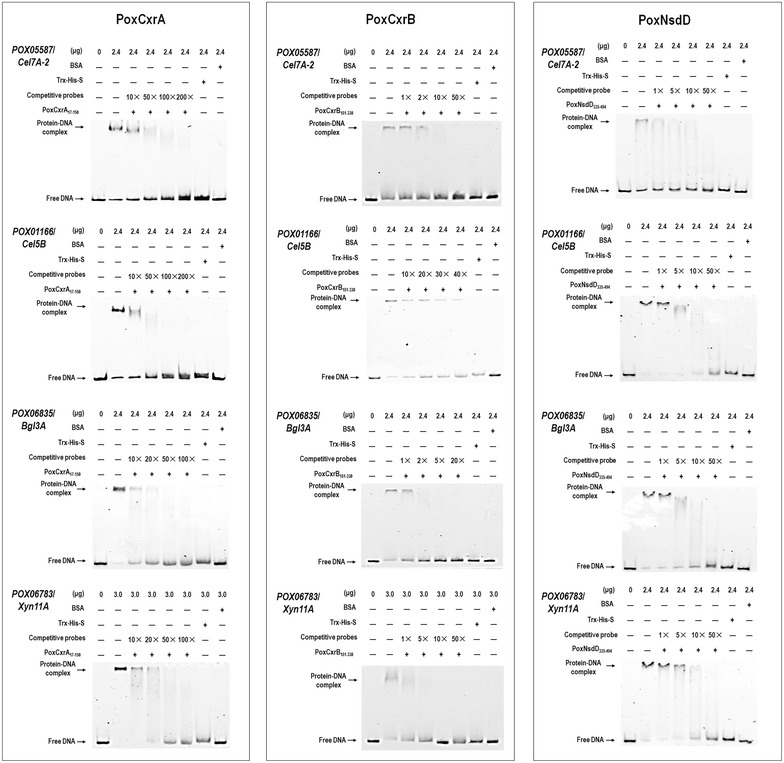
Electrophoretic mobility shift assay showing the interactions between the DNA-binding domains of the regulators and the promoter sequences of cellulase and xylanase genes

To confirm the regulation of the genes targeted by PoxCxrA, PoxCxrB, and PoxNsdD, in vitro binding experiments were performed with an electrophoretic mobility shift assay (EMSA). The thioredoxin (TRX)–His–S-tagged putative DNA-binding domains of PoxCxrA, PoxCxrB, and PoxNsdD, designated PoxCxrA_17–150_, PoxCxrB_181–330_, and PoxNsdD_335–494_, respectively, were individually recombinantly expressed in *Escherichia coli* Rossetta, and purified. The putative protein-binding DNA fragments (100–500 bp) in the promoter regions of the targeted genes, including the major cellulase and xylanase genes *POX05587/Cel7A*-*2*, *POX01166/Cel5B*, *POX06835*/*Bgl3A*, and *POX06783*/*Xyn11A*, were tagged with 6-carboxyfluorescein (FAM). Shifted bands appeared in all the EMSA gels when PoxCxrA_17–150_, PoxCxrB_181–330_, or PoxNsdD_335–494_ was mixed individually with the FAM-tagged DNA fragments corresponding to the promoter regions of these genes. The binding strengths increased dramatically as the amount of protein was increased from 1.2 to 4.8 μg (Additional file 10: Figure S6). Competitive experiments were also performed using protein-binding DNA fragments lacking the FAM label as competitive probes. The concentrations of the shifted bands gradually decreased as the amount of competitive probe increased. However, no shifted band was detected between any DNA fragment and the high-concentration TRX–His–S fusion protein purified from the total proteins of *E*. *coli* cells containing the empty vector or bovine serum albumin (BSA) protein, which were used as the negative controls (Fig. 7). These results suggest that PoxCxrA_17–150_, PoxCxrB_181–330_, and PoxNsdD_335–494_ specifically bound all the tested DNA sequences from the promoter regions of the major cellulase and xylanase genes in vitro.

**Fig. 8:**
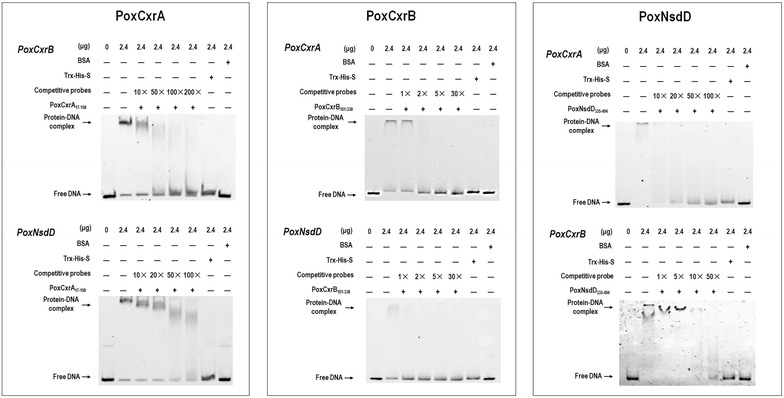
Electrophoretic mobility shift assay showing the interactions between the DNA-binding domains of the regulators and the promoter sequences of the newly identified regulatory genes

EMSA experiments were also performed to evaluate the interactions between the newly identified regulatory genes. The promoter regions of genes *PoxCxrB* and *PoxNsdD* were used as the probes to test their binding by PoxCxrA_17–150_; those of *PoxCxrA* and *PoxNsdD* were used to test their binding by PoxCxrB_181–330_; and those for *PoxCxrA* and *PoxCxrB* were used to test their binding by PoxNsdD_335–494_. PoxCxrA_17–150_, PoxCxrB_181–330_, and PoxNsdD_335–494_ specifically bound all the tested DNA sequences from the promoter regions of the newly identified regulatory genes in vitro (Fig. 8 and Additional file 11: Figure S7).

## Discussion

In this study, 10 novel regulators of cellulase and xylanase gene expression in *P*. *oxalicum* were identified with transcriptomic profiling and genetic analyses. Among the 10 novel genes, the deletion mutants of *PoxCxrA*, *PoxCxrB*, and *PoxNsdD* genes were confirmed by PCR using gene-specific primers, Southern hybridization analysis and gene complementation. The cellulase and xylanase production of the complemented strains C∆*PoxCxrA*, C∆*PoxCxrB* and C∆*PoxNsdD* could not be fully restored to the levels of the parent strain ∆*PoxKu70*, which might result from different transcription levels of the complementary genes from that in ∆*PoxKu70*. The complementary genes *PoxCxrB* and *PoxNsdD* were introduced into the locus of an aspartic protease gene *PoxPepA* (*POX05007*) whose deletion did not cause any alterations in cellulase and xylanase production in ∆*PoxKu70* (Jia-Xun Feng et al., unpublished), while the complementary cassette of *PoxCxrA* might randomly integrate into unknown locus in the genome. The knock-out of genes *PoxCxrA*, *PoxCxrB*, and *PoxNsdD* did not affect the vegetative growth of *P*. *oxalicum* in the presence of glucose, but very significantly affected the utilization of cellulose, indicating their important roles in cellulose degradation. The functions of PoxCxrA and PoxCxrB have not been reported in the literature until now.

PoxNsdD is a homologue of NsdD in *Aspergillus*. NsdD functions as an activator of sexual development [30] and a key repressor of asexual development in *Aspergillus* in specific environments [29, 31]. NsdD represses conidiation by downregulating the expression of *BrlA*, which encodes a key TF essential for conidiophore development in *Aspergillus* [30]. NsdD also functions downstream from fluffy gene G (*FluG*), but upstream from *BrlA*. In *P*. *oxalicum* 114-2, *FluG* deletion results in neither the fluffy phenotype nor any change in conidiation, and *BrlA* is required but not sufficient for conidiation, suggesting that the mechanism of conidiophore development differs in *P*. *oxalicum* and *A*. *nidulans* [27]. However, NsdD has not yet been ascribed any regulatory function regarding cellulase and xylanase gene expression in the filamentous fungi.

Interestingly, in *P*. *oxalicum* HP7-1, the deletion of *PoxCxrB* and *PoxNsdD* reduced the production of CBH, endo-β-1,4-glucanase (EG), and xylanase, but increased BGL production. BGL is responsible for the degradation of cellobiose to glucose, and cellobiose is a major inducer of cellulase gene expression. Therefore, an increase in BGL activity might reduce the amount of cellobiose, thus reducing the expression of the cellulase genes. Surprisingly, in Δ*PoxCxrB* and Δ*PoxNsdD*, the transcription level of the *bgl* gene *POX06835*/*Bgl3A*, which is thought to encode a predominantly extracellular BGL [32], was downregulated relative to its expression in Δ*PoxKu70*. However, the total extracellular BGL activities in the mutants were higher than that in Δ*PoxKu70*, suggesting that other extracellular BGLs exist.

The knock-out of the known regulatory gene *PoxClrC* caused no significant changes in FPase activity 6 days after inoculation onto Avicel compared with that in the parental strain Δ*PoxKu70*. However, its deletion significantly affected EG and/or BGL production (Jia-Xun Feng et al., unpublished observation). In a previous study, the BGL deletion mutant showed reduced cellulase production when cultured on Avicel medium after transfer from glucose medium [28]. These discrepancies may be attributable to different genetic backgrounds, different fungal culture conditions, and/or different methods of measuring enzyme production in the different studies.

**Fig. 9:**
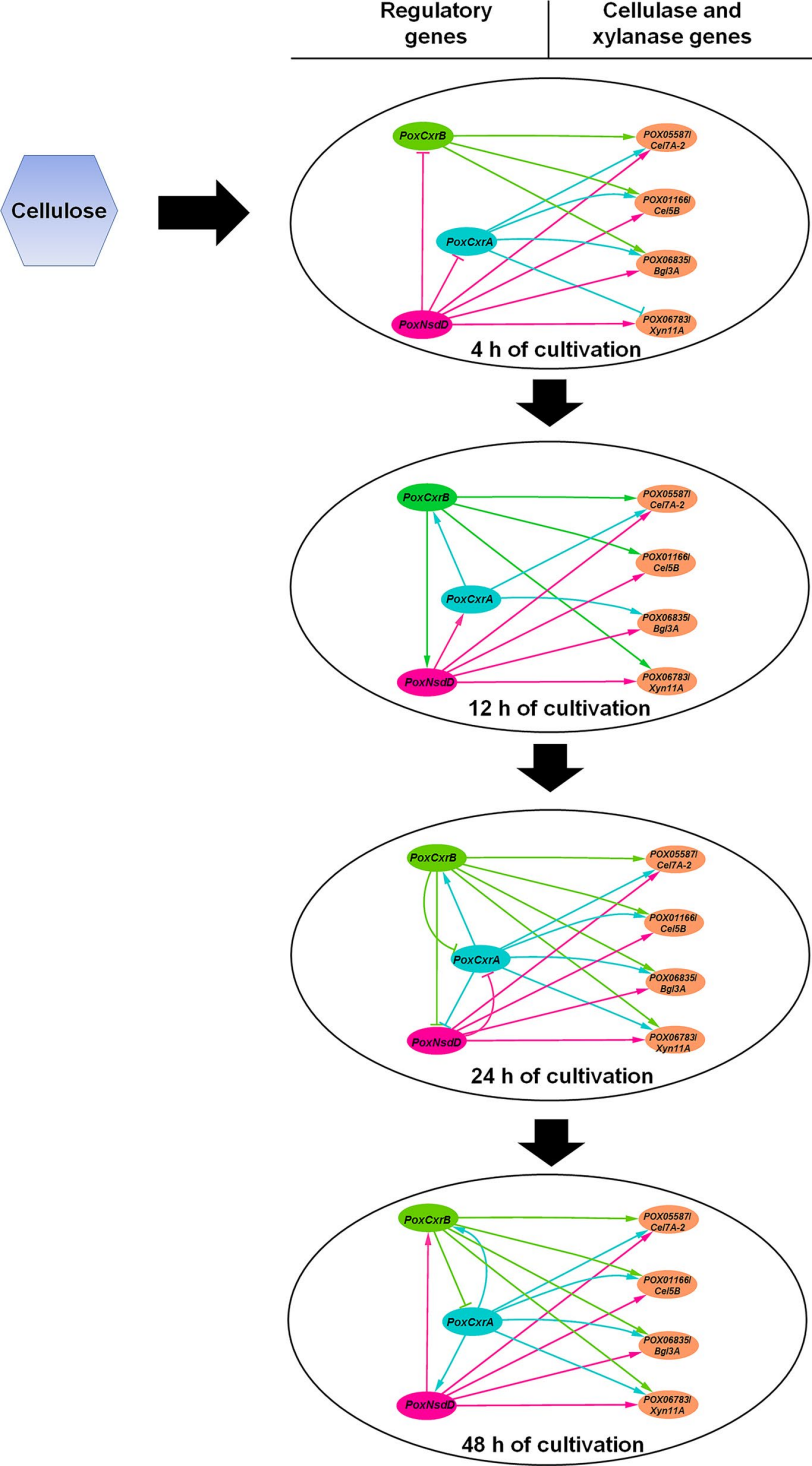
Regulation network of the three novel key regulatory genes *PoxCxrA, PoxCxrB*, and *PoxNsdD* identified in *P. oxalicum*. Lines with arrows represent activation and barred lines represent inhibition. Green, blue, and purple lines show regulation by *PoxCxrA*, *PoxCxrB*, and *PoxNsdD*, respectively

Based on the experimental data generated in this study, we propose a novel real-time regulatory network of genes that controls the expression of the cellulase and xylanase genes of *P*. *oxalicum* in the presence of cellulose (Fig. 9). In the early stage of induction (4–12 h) of cellulose, *PoxNsdD* represses the expression of *PoxCxrA* and *PoxCxrB*. Subsequently, *PoxNsdD* stimulates *PoxCxrA* expression, and *PoxCxrA* induces *PoxCxrB* expression. Interestingly, *PoxCxrB* also enhances the transcription of *PoxNsdD*. By contrast, the regulatory relationships between these novel regulatory genes become more complex in the later period (24–48 h) of induction. At 24 h, *PoxCxrA* increases the expression of *PoxCxrB*, and suppressing the expression of *PoxNsdD*, whose expression is also repressed by both *PoxCxrB* and *PoxNsdD*. Moreover, *PoxCxrB* reduces the transcription of *PoxNsdD*. As at 24 h, *PoxCxrA* increases the transcription of genes *PoxCxrB* and *PoxNsdD*, whose expression is repressed by *PoxCxrB* at 48 h, whereas *PoxNsdD* increases the expression of *PoxCxrB* at this time (Fig. 9).

During the whole period of induction, all three novel regulatory genes *PoxCxrA*, *PoxCxrB*, and *PoxNsdD* play positive roles in regulating the expression of the major cellulase and xylanase genes, including *POX05587*/*Cel7A*-*2*, *POX01166*/*Cel5B*, *POX06835*/*Bgl3A*, and *POX06783*/*Xyn11A*, except for the regulation of *POX01166*/*Cel5B*, *POX06783*/*Xyn11A*, and *POX06835*/*Bgl3A* by *PoxCxrA* and *PoxCxrB* at 4 or 12 h. Overall, the newly identified regulatory genes *PoxCxrA*, *PoxCxrB*, and *PoxNsdD* regulate the expression of one another and directly regulate the expression of the cellulase and xylanase genes. However, the regulatory mechanism changes dynamically during the growth of fungal cells in the presence of cellulose (Fig. 9).

A protein alignment indicated that the three newly identified regulators, PoxCxrA, PoxCxrB, and PoxNsdD, are conserved in most filamentous ascomycete fungi capable of degrading cellulose, such as *Aspergillus*, *Talaromyces*, *Trichoderma*, and *Neurospora*. These data suggest that the three proteins and their homologues might play important roles in the regulation of cellulase and xylanase gene expression in ascomycete fungi, but this idea requires further confirmation.

## Conclusions

In this study, our data show that *PoxCxrA, PoxCxrB*, and *PoxNsdD* are essential genes in the regulation of cellulase and xylanase gene expression in *P*. *oxalicum*. More importantly, *PoxCxrA*, *PoxCxrB*, and *PoxNsdD* regulate the expression of one another and directly regulate the expression of the cellulase and xylanase genes. These findings provide novel insights into the regulation of fungal cellulase and xylanase gene expression and provide a basis for genetically engineering or breeding fungi for the hyperproduction of cellulases and xylanases.

## Methods

### *Penicillium oxalicum* strains and culture conditions

The *P. oxalicum* strains used in this study were maintained on potato–dextrose agar (PDA) plates at 4 °C. The fungal spores used for reproduction were harvested after 6 days on PDA plates at 28 °C, resuspended in buffer containing 0.1% Tween 80, and adjusted to a concentration of 1 × 10^8^ per milliliter.

For the transcriptomic (RNA-Seq) analysis of gene expression in *P*. *oxalicum* strain HP7-1 (China General Microbiological Culture Collection [CGMCC] no. 10781), 1 mL of spore suspension (1.0 × 10^8^/mL) was used to inoculate 100 mL of modified minimal medium (MMM [g/L]: (NH_4_)_2_SO_4_ 4.0, KH_2_PO_4_ 4.0, CaCl_2_ 0.6, MgSO_4_·7H_2_O 0.60, FeSO_4_·7H_2_O 0.005, MnSO_4_ 0.0016, ZnCl_2_ 0.0017, CoCl_2_ 0.002, and 1 mL of Tween 80) containing glucose (1.0 g), wheat bran (4.0 g) (purchased from a local farmer’s market in Nanning, China), or wheat bran (4.0 g) plus Avicel^®^ PH-101 (2.0 g) (Cat No. 11365-1KG; Sigma-Aldrich, Darmstadt, Germany) as the sole carbon source, respectively. The inoculated cultures were placed in a shaker at 28 °C, and shaken at 180 rpm for 72 h. Mycelia were collected from the cultures with four-layer filter fabrics, and washed with diethyl-pyrocarbonate-treated water before RNA extraction.

The constructed *P*. *oxalicum* mutants of the candidate TF genes were inoculated into 100 mL of MMM containing a final concentration of 2% (w/v) Avicel. The inoculated medium was incubated at 28 °C, with shaking at 180 rpm for 6 days. The supernatant was collected by centrifugation at 11,300×*g* for 10 min to assay the FPase activity.

To measure the growth profiles, Δ*PoxCxrA* (Δ*POX01167*, CGMCC no. 12965), Δ*PoxCxrB* (Δ*POX04420*, CGMCC no. 12966), Δ*PoxNsdD* (Δ*POX08415*, CGMCC no. 12967), and the parental strain Δ*PoxKu70* (CGMCC no. 3.15650) [5] were inoculated into MMM containing 1% (w/v) glucose or 2% (w/v) Avicel and incubated at 28 °C with shaking at 180 rpm. The mycelia were collected every 12 h until 72 h.

A shift experiment was performed followed by an RT-qPCR analysis of gene expression and an enzymatic activity assay of Δ*PoxCxrA*, Δ*PoxCxrB*, Δ*PoxNsdD*, and the parental strain Δ*PoxKu70* [5]. Approximately 1.0 × 10^8^/mL spores from the *P*. *oxalicum* mutant strains were grown in 100 mL of MMM containing 1% (w/v) glucose at 28 °C with shaking at 180 rpm for 24 h. Equal portions of the harvested and washed mycelial samples were then aseptically inoculated into fresh MMM containing 2% (w/v) Avicel as the sole carbon source. After culture for 4, 12, 24, or 48 h, the mycelia were harvested, and the total RNA was extracted for RT-qPCR.

For the enzymatic activity assay of the three mutants Δ*PoxCxrA*, Δ*PoxCxrB*, and Δ*PoxNsdD* under induction conditions, the pregrown mycelia of these strains were cultured for 2–4 days in MMM containing Avicel as the sole carbon source, as described above.

### Total DNA and RNA extraction

Total DNA and RNA were extracted with chemical methods, as previously described [5]. For total DNA extraction, the harvested mycelia were first washed with sterile water, ground in liquid nitrogen, and then added to a specific amount of lysate reagent (40 mM Tris–HCl, 10 mM ethylenediaminetetraacetic acid, 20 mM sodium acetate, and 1% sodium dodecyl sulfate; pH 8.0) in a ratio of 1 mL of lysate reagent/100 mg of mycelium powder. The total DNA was separated by centrifugation at 11,300×*g* for 10 min.

For total RNA extraction, the TRIzol RNA Kit (Life Technologies, Carlsbad, CA, USA) was used, according to the manufacturer’s instructions. The concentration and integrity of the extracted total RNA were assessed by the absorbance at 260 nm (A_260_) and the A_260_/A_280_ ratio, respectively, and with electrophoresis on 1% agarose gel.

### Construction of gene deletion mutants of *P*. *oxalicum*

Gene deletion mutants of *P*. *oxalicum* were constructed with the homologous recombination method, reported by Zhao et al. [5]. Protoplasts of the parental strain Δ*PoxKu70* were first prepared. Fresh conidia of Δ*PoxKu70* were cultured at 28 °C with shaking at 180 rpm for 8 h in 200 mL of CM medium (g/L: NaNO_3_ 30, KCl 2.60, MgSO_4_·7H_2_O 2.60, KH_2_PO_4_ 7.60, d-glucose 10.0, peptone 2.0, yeast extract 1.0, and acid-hydrolyzed casein 1.0; pH 6.5). The grown mycelia were collected, washed three times with 0.6 M MgSO_4_·7H_2_O, and lysed for 2.5 h in OM solution (1.2 M MgSO_4_·7H_2_O, 10 mM NaH_2_PO_4_, 6 g/L snailase, 4 g/L lysozyme, and 6 g/L lysing enzymes from *T*. *harzianum* [Sigma-Aldrich, St. Louis, MO, USA]; pH 5.8). After the addition of trapping buffer (0.4 M sorbitol, 0.1 M Tris–HCl; pH 7.0), the protoplasts were collected by centrifugation, precipitated, and washed sequentially with 1 M sorbitol and STC solution (1 M sorbitol and 0.1 M Tris–HCl, and 0.1 M CaCl_2_; pH 8.0). The protoplasts were finally resuspended in 0.5 mL of 4 × STC and 1 × PTC (40% polyethylene glycol 3350, 0.1 M Tris–HCl, and 0.1 M CaCl_2_; pH 8.0), and stored at − 80 °C until analysis.

The knock-out cassette for each candidate gene was constructed with fusion PCR, and included a 1.8-kb DNA fragment encoding the G418-resistance gene and approximately 2 kb each of the upstream and downstream DNA fragments flanking the target gene. A specific amount of each knock-out cassette was dissolved in 0.1 M spermidine, added to the protoplast suspension, and incubated on ice for 30 min. After incubation, the mixture was transferred to 1 × PTC solution and maintained at room temperature for 25 min. The cultured PTC solution was then added to Petri dishes, mixed with OCM medium containing 1.0 g/L casein enzymatic hydrolysate, 1.0 g/L yeast extract, 273.6 g/L sucrose, and 10.0 g/L agar, and incubated at 50 °C for 30 min. PDA medium containing 500 μg/mL G418 and 250 μg/mL hygromycin B was added to the surface of the OCM medium. After 5 days in culture at 28 °C, the transformants were selected and purified.

### Gene complementation

To construct the complemented strains of the mutants ∆*PoxCxrA*, ∆*PoxCxrB* and ∆*PoxNsdD*, complementary cassettes carrying Bleomycin resistance gene were, respectively, constructed, comprised of the upstream sequence of the integrative locus for the complementary cassette, the coding sequence of the complementary gene, the Bleomycin resistance gene and the downstream sequence of the integrative locus for the complementary cassette.

For constructing the complementary *PoxCxrA* cassette, an approximately 5.2 kb DNA including the upstream sequence of the gene *PoxCxrA* containing the promoter region, the coding sequence of *PoxCxrA* and 0.4 kb of putative terminator of *PoxCxrA* was obtained via PCR using the specific primer pair CxrA-L-F/CxrA-R. The Bleomycin resistance gene was amplified from the plasmid pZTBle stored in our laboratory using primer pair Ble-F/Ble-R. An approximately 2.5 kb DNA containing the 3′-downstream sequence of *PoxCxrA* was amplified from genome of *P*. *oxalicum* HP7-1 using the primer pair CxrA-R-F/CxrA-R-R. The three PCR fragments were ligated using fusion PCR, and then the complementary *PoxCxrA* cassette was amplified using the nest primer pair C-CxrA-F/C-CxrA-R (Additional file 7: Table S4). The resultant cassette was introduced into the ∆*PoxCxrA* mutant to generate the complementary strain C∆*PoxCxrA*.

The complementary strains C∆*PoxCxrB* and C∆*PoxNsdD* were constructed similarly as described above. Four DNA fragments, including the left-flanking and right-flanking sequences of an aspartic protease gene *PoxPepA* (*POX05007*) in the genome of *P*. *oxalicum* HP7-1, Bleomycin resistance gene and the complementary gene, were amplified using primer pairs PepA-L-F/PepA-L-R, PepA-R-F/PepA-R-R, Ble-F/Ble-R and CxrB-F/CxrB-F (or NsdD-F/NsdD-R), respectively (Additional file 7: Table S4). The four PCR products were ligated using *pEASY* ^®^-Uni Seamless Cloning and Assembly Kit (TransGen Biotech, Beijing, China). Complementary cassettes of *PoxCxrB* and *PoxNsdD* were then generated by PCR using primer pair C-PepA-F/C-PepA-R (Additional file 7: Table S4), and, respectively, integrated into the locus of *PoxPepA* in genome of the corresponding deletion mutants.

### RNA sequencing

RNA was sequenced with the method described by Zhao et al. [5]. A cDNA library of each sample was constructed for RNA-seq and then assessed with an Agilent 2100 Bioanalyzer (Agilent Technologies, Santa Clara, CA, USA) and the ABI StepOnePlus Real-Time PCR System (Applied Biosystems, Foster City, CA, USA). The Illumina HiSeq 2000 system was used to sequence the cDNA libraries. The raw reads generated were filtered, and the following reads were removed: those including the adapter sequence, those with a high content (> 10%) of unknown bases, and low-quality reads in which the percentage of low-quality bases (*Q* ≤ 10) was > 50%. The clean reads thus generated were then subjected to quality control (QC) through drawing the base compositions and quality distributions. After QC, the clean reads were mapped onto the genome of *P*. *oxalicum* wild-type strain HP7-1 to search for gene homologies and their functional annotations, using the software BWA v0.7.10-r789 [22] and Bowtie2 v2.1.0 [23]. The RSEM v1.2.12 software [24] was used to analyze the gene expression levels (fragments per kilobase of exon per million mapped reads, FPKM). The differentially expressed genes were screened with the NOISeq tool [25], with |log_2_ fold change| > 0.8 and probability ≥ 0.8 as thresholds. The reliability of RNA-Seq was assessed in three biological replicates of each sample with Pearson’s correlation coefficient. These datasets (FPKM) were subjected to a hierarchical cluster analysis using the software Heml 1.0 [33] to determine the groups of genes with similar expression patterns for a different group of regulons. BLAST v2.2.26 (http://blast.ncbi.nlm.nih.gov/Blast.cgi) was used for gene homology and function annotation. The differently expressed genes detected by comparative assays were functionally analyzed based on Kyoto encyclopedia of genes and genomes (KEGG) annotation to Genome of *P*. *oxalicum* HP7-1 [5].

### Southern hybridization analysis

The mutants were analyzed with Southern hybridization, as previously described [5]. The genomic DNA of each gene deletion mutant was digested with Apa1 for *PoxCxrA*, BamH1 for *PoxCxrB*, and EcoR1 for *PoxNsdD* (TakaRa Bio Inc., Dalian, China). The enzyme-digested DNA fragments were separated on 0.8% agarose gel and transferred to Hybond-N^+^ nylon membrane (GE Healthcare Limited, Buckinghamshire, UK). The probes amplified with the indicated primers (Additional file 7: Table S4), using the genomic DNA of HP7-1 as the template, were labeled and detected with the DIG-High Prime DNA Labeling and Detection Starter Kit (Life Technologies, Carlsbad, CA, USA).

### Real-time RT-qPCR

RT-qPCR was used to compare the expression levels of the cellulase and xylanase genes in the deletion mutants and the parental strain Δ*PoxKu70* based on a previously described method [5]. The PrimeScript RT Reagent Kit (TakaRa Bio Inc.) was used to synthesize the first-stand cDNA. The final RT-qPCR mixture (20 μL) contained 0.8 μL of 10 μM primers (Additional file 7: Table S4), 0.2 μL of first-stand cDNA as the template, and 10 μL of SYBR Premix Ex Taq II (TakaRa Bio Inc.). All the reactions were run for 40 cycles of 95 °C for 3 s and 60 °C for 30 s. The fluorescent signal was detected at the end of each extension step at 80 °C. The relative expression of the target genes was calculated using the actin gene (*POX09428*) as the control and normalizing all expression to the parental strain Δ*PoxKu70*. All RT-qPCRs were repeated independently at least three times.

### Expression of DNA-binding-domain-encoding sequences of *PoxCxrA*, *PoxCxrB*, and *PoxNsdD,* and purification of the recombinant polypeptides

The DNA-binding-domain-encoding sequences of *PoxCxrA*, *PoxCxrB*, and *PoxNsdD* were expressed in *E. coli* Rossetta (Transgen Biotech, Beijing, China). The DNA fragments encoding the DNA-binding domains of PoxCxrA (amino acids 17–150), PoxCxrB (amino acids 181–330), and PoxNsdD (amino acids 335–494) were amplified with PCR using the cDNA of *P*. *oxalicum* HP7-1 as the template, with primer pairs PoxCxrA_17–150_-F/PoxCxrA_17–150_-R, PoxCxrB_181–330_-F/PoxCxrB_181–330_-R, and PoxNsdD_335–494_-F/PoxNsdD_335–494_-R, respectively (Additional file 7: Table S4). The PCR products were digested with *Eco*R1 and/or *Not*1, and *Bam*H1, and then inserted into the expression vector pET-32a(+) digested with the corresponding restriction endonucleases. The fusion plasmids were introduced into competent *E*. *coli* Rossetta cells with chemical transformation. After PCR confirmation, the recombinant strains were cultured in Luria–Bertani medium for 8 h, and then induced with 0.5 mM isopropyl-β-d-thiogalactopyranoside at 25 °C to produce recombinant proteins fused with TRX, His, and S tags. The strain containing the empty vector pET-32a(+) was cultured as the control.

After the cells were disrupted with ultrasonication, the fusion proteins were purified with affinity chromatography on TALON Metal Affinity Resin (Clontech, Palo Alto, CA, USA), according to the instructions of the manufacturer. The protein concentrations were measured with the Bradford Protein Assay Kit (Tiangen, Beijing, China), according to the manual.

### EMSA

DNA fragments (100–500 bp upstream from the ATG start codons of *PoxCxrA*, *PoxCxrB*, *PoxNsdD*, *POX01166/Cel5B*, *POX05587/Cel7A*-*2*, *POX06835*/*Bgl3A*, and *POX06783*/*Xyn11A*) were generated with PCR and the corresponding primers (Additional file 7: Table S4). Each reverse primer was labeled with FAM at its 3′-terminus. After purification, 40 ng of each FAM-labeled PCR product was mixed with various amounts (0–4.8 μg) of DNA-binding-domain polypeptide of the regulator proteins in a binding buffer (0.1 mg/mL BSA, 20 mM Tris–HCl [pH 8.0], 5% glycerol, 50 mM KCl, 1 mM dithiothreitol). For the competitive binding experiments, a known amount of binding protein was mixed with various amounts of probe under the conditions described above. The protein–DNA complexes were separated with 4% polyacrylamide–Tris–acetic acid–EDTA (TAE) gel electrophoresis, and visualized with the Bio-Rad ChemiDoc™ MP Imaging System (Bio-Rad Laboratories, Inc. Hercules, CA, USA) at an excitation wavelength of 489–506 nm. Each protein–DNA complex was retarded relative to the free DNA. BSA alone or TRX–His–S purified from the total protein extracted from *E*. *coli* cells containing the empty vector pET-32a(+) was used as the negative control.

### Enzyme activities and protein concentrations

The cellulase and xylanase activities and protein concentrations were measured as described previously [5]. Briefly, FPase activity was measured with Whatman No. 1 filter paper (50 mg, 1.0 × 6.0 cm^2^) (GE Healthcare Company) as the substrate in 1.0 mL of 100 mM citrate buffer (pH 5.0) and 0.5 mL of suitably diluted crude cellulase for 1 h at 50 °C. CMCase activity was tested by incubating 1.0% CMC-Na (Sigma-Aldrich, Darmstadt, Germany) solution in citrate buffer (100 mM, pH 5.0) containing a specific amount of crude cellulase for 30 min at 50 °C. Xylanase activity was measured under similar conditions, except that the substrate was replaced with 1.0% xylan from beechwood (Megazyme International Ireland, Wicklow, Ireland), and the incubation time was 10 min. After two volumes of 3,5-dinitrosalicyclic acid were added, the reducing sugars generated were measured at 540 nm. One unit of enzymatic activity (U) was defined as the amount of enzyme required to produce 1 μmol of reducing sugar per min from the reaction substrates. Triplicate independent experiments were performed for each sample.

To measure the pNPCase and pNPGase activities, *p*-nitrophenyl-β-d-cellobioside (pNPC) and *p*-nitrophenyl β-d-glucopyranoside (pNPG) (Sigma-Aldrich) were used as the substrates, respectively, liberating *p*-nitrophenol at 50 °C and pH 5.0 after incubation for 15 min in 140 μL reaction systems (116 μL of 100 mM citrate buffer, 14 μL of 25 mM substrate, and 10 μL of diluted crude enzyme). Sodium carbonate (0.4 M, 70 μL) was used to stop the reactions. The *p*-nitrophenol produced was measured with spectrometry at 410 nm. One unit of enzymatic activity (U) was defined as the amount of enzyme that produced 1 μmol of *p*-nitrophenol per min from the appropriate substrate. Each sample was analyzed independently at least three times.

The protein concentrations were measured with a Pierce™ detergent compatible Bradford assay kit (Pierce Biotechnology, Rockford, IL, USA), according to the manufacturer’s instructions.

### Phylogenetic analysis

The amino acid sequences of PoxCxrA, PoxCxrB, and PoxNsdD homologues were downloaded from the NCBI BlastP website (https://blast.ncbi.nlm.nih.gov/Blast.cgi). A phylogenetic tree was constructed using the MEGA version 7.0 software [34] with the neighbor-joining method and a Poisson correction model. In this process, 1000 replicates were used to calculate the bootstrap values.

### Statistical analysis

Student’s *t* test (two-tailed) in Microsoft Excel (Office 2016) (Microsoft, Redmond, WA, USA) was used for the statistical analysis of the data.

### Accession numbers

All transcriptomic data are available from the Sequence Read Archive database (Accession Number SRA505232). DNA sequences are available from the GenBank database (Accession Numbers KY368171–KY368173, KY922971, and KY860734–KY860740).

## Additional files

**Additional file 1: Figure S1.** Cellulase activity of *P. oxalicum* HP7-1 in the presence of glucose (Glu), wheat bran (WB), or wheat bran and Avicel (WA). Data are the means of three biological replicates.

**Additional file 2: Table S1.** Summary of RNA-sequencing reads obtained for *P. oxalicum* strain HP7-1 and its derived mutants.

**Additional file 3: Figure S2.** Pearson’s correlation analysis of the transcriptomes of *P. oxalicum* HP7-1 in the presence of glucose (Glu), wheat bran (WB), or wheat bran and Avicel (WA) as the carbon source. RNA for sequencing was extracted from cells sampled 72 h after inoculation.

**Additional file 4: Table S2.** List of 108 genes differentially coexpressed on all carbon sources tested, including glucose (Glu), wheat bran (WB), and wheat bran and Avicel (WA).

**Additional file 5: Table S3.** Forty candidate transcription factors regulating the expression of cellulase and xylanase genes in *P. oxalicum* detected with a transcriptomic profiling analysis.

**Additional file 6: Figure S3.** Confirmation analysis of the deletion mutants of 31 candidate genes derived from the parental strain Δ*PoxKu70*. (A–AE) PCR analysis of: (A) Δ*POX00864*; (B) Δ*PoxClrC*; (C) Δ*POX01167/PoxCxrA*; (D) Δ*POX01183*; (E) Δ*POX01184*; (F) Δ*POX02261*; (G) Δ*POX02682*; (H) Δ*POX02944*; (I), Δ*POX03888*; (J) Δ*POX03910*; (K) Δ*POX04193*; (L) Δ*POX04420/PoxCxrB*; (M) Δ*POX04590*; (N) Δ*POX04676*; (O) Δ*POX04772*; (P) Δ*POX04860*; (Q) Δ*POX05374*; (R) Δ*POX05436*; (S), Δ*POX05726*; (T) Δ*POX06377*; (U) Δ*POX06396*; (V) Δ*POX06425*; (W) Δ*PoxBrlA*; (X) Δ*POX06759*; (Y) Δ*PoxFlbD*; (Z) Δ*POX07934*; (AA) Δ*POX08415/PoxNsdD*; (AB) Δ*POX08702*; (AC) Δ*POX08910*; (AD) Δ*POX09356*; and (AE) Δ*POX09460*. M, 1-kb DNA marker; lanes 1–3, three transformants constructed for each candidate gene; lane 4, Δ*PoxKu70*; lane 5, ddH_2_O. (AF–AH) Southern hybridization analysis: (AF) Δ*POX01167/PoxCxrA*; M, 1-kb DNA marker; lane 1, Δ*PoxKu70*; lane 2, Δ*POX01167/PoxCxrA*-6; lane 3, Δ*POX01167/PoxCxrA*-9; lane 4, Δ*POX01167/PoxCxrA*-11. (AG) Δ*POX04420*; M, 1-kb DNA marker; 1, Δ*PoxKu70*; 2, Δ*POX04420/PoxCxrB*-2; 3, Δ*POX04420/PoxCxrB*-6; 4, Δ*POX04420/PoxCxrB*-8. (AH) Δ*POX08415*; M, 1-kb DNA marker; lane 1, Δ*PoxKu70*; lane 2, Δ*POX08415/PoxNsdD*-1; lane 3, Δ*POX08415/PoxNsdD*-5; lane 4, Δ*POX08415/PoxNsdD*-9.

**Additional file 7: Table S4.** Primers used in this study.

**Additional file 8: Figure S4.** Unrooted phylogenetic tree of PoxCxrA, PoxCxrB, and PoxNsdD and their putative homologues. The dendrogram was constructed with the MEGA 7 software using the neighbor-joining method and a Poisson model. Bootstrap values shown at the nodes were derived with 1000 replicates, and the branch lengths, which correspond to the divergence of the sequences, are indicated by the scale bar.

**Additional file 9: Figure S5.** Confirmation analysis of the complementary strains. Targeted complementary genes including *PoxCxrA*, *PoxCxrB* and *PoxNsdD* were amplified using primer pairs CxrA-CDS-F/CxrA-CDS-R, CxrB-CDS-F/CxrB-CDS-R and NsdD-CDS-F/NsdD-CDS-R. Bleomycin resistance gene was amplified using primer pair Ble-F/Ble-R. M, 1-kb DNA marker; lane 1, complementary strain; lane 2, Δ*PoxKu70*; lane 3, corresponding deletion mutant strain.

**Additional file 10: Figure S6.** Electrophoretic mobility shift assay showing the interaction between the DNA-binding domains of the regulators and the promoter sequences of cellulase and xylanase genes. The experiments were performed without competitive probes.

**Additional file 11: Figure S7.** Electrophoretic mobility shift assay showing the interaction between the DNA-binding domains of the regulators and the promoter sequences of the newly identified regulatory genes. The experiments were performed without competitive probes.

## Abbreviations

CWDEs: plant-cell-wall-degrading enzymes
EMSA: electrophoretic mobility shift assay
Glu: glucose
HMG: high-mobility group
PDA: potato-dextrose agar
RT-qPCR: quantitative reverse transcription-PCR
TF: transcription factor
TRX: thioredoxin
WA: wheat bran plus Avicel
WB: wheat bran
CMCase: carboxymethylcellulase
FPase: filter-paper cellulase
pNPCase: *p*-nitrophenyl-β-cellobiosidase
pNPGase: *p*-nitrophenyl-β-glucopyranosidase
EG: endo-glucanase
CBH: cellobiohydrolase
BGL: β-glucosidase
MMM: modified minimal medium
FPKM: fragments per kilobase of exon per million mapped fragments
