# Supplementary figures and images for: Transcriptomic profiling and genetic analyses reveal novel key regulators of cellulase and xylanase gene expression in Penicillium oxalicum
Source: Biotechnol Biofuels. 2017 Nov 22;10:279. doi: 10.1186/s13068-017-0966-y (PMC5700522; doi:10.1186/s13068-017-0966-y)

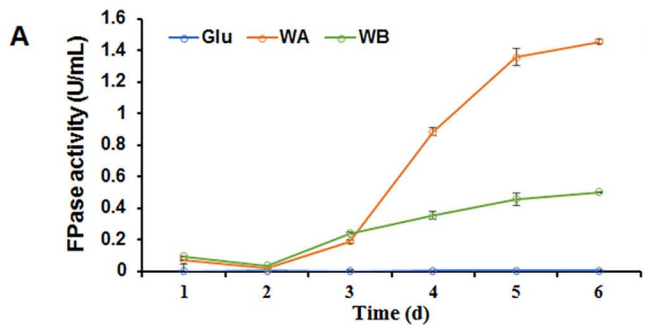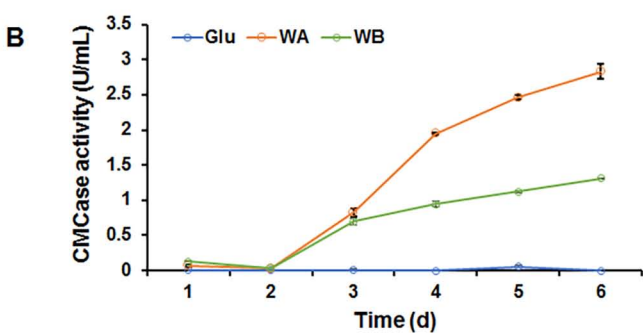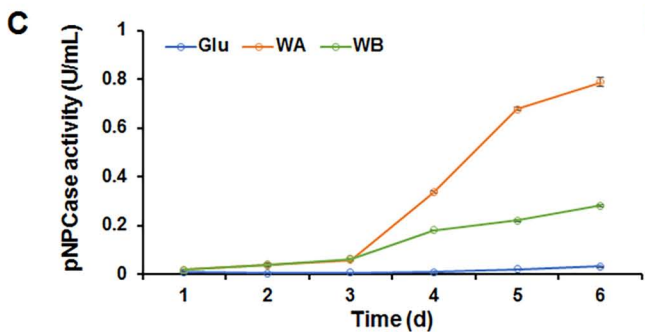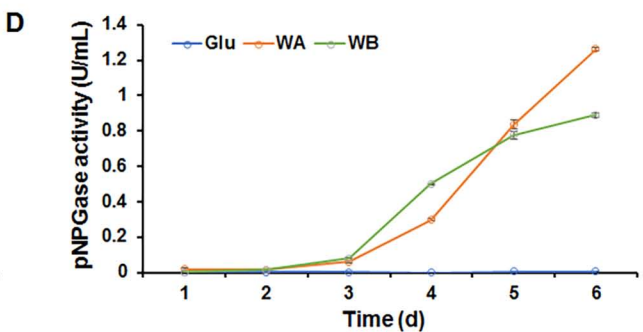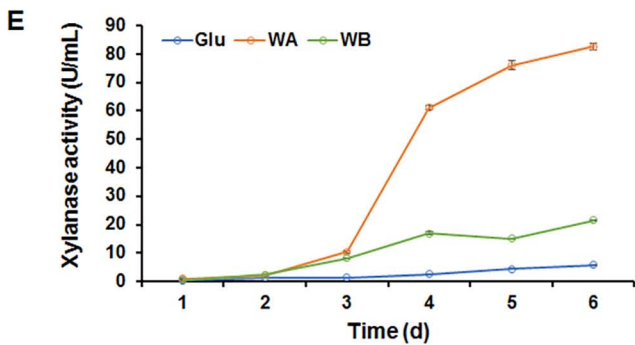

Supplement: Supplementary file 1 — Additional file 1: Figure S1. Cellulase activity of P. oxalicum HP7-1 in the presence of glucose (Glu), wheat bran (WB), or wheat bran and Avicel (WA). Data are the means of three biological replicates. [file 13068_2017_966_MOESM1_ESM.pdf]

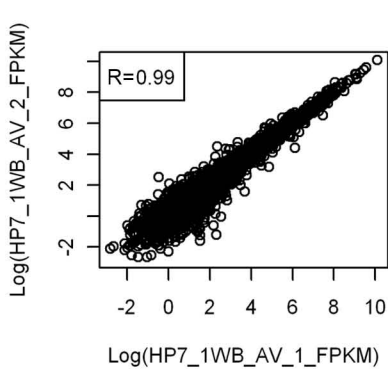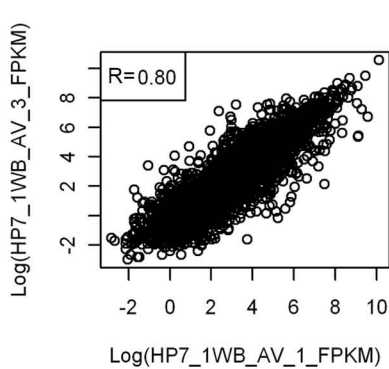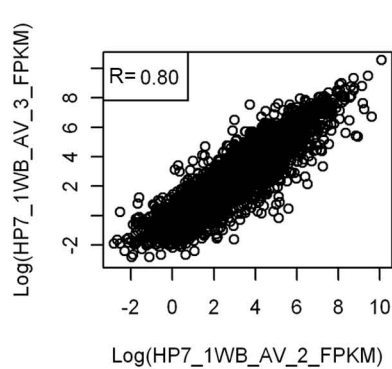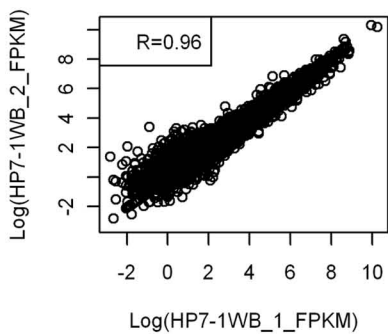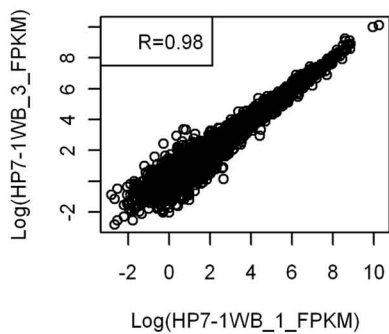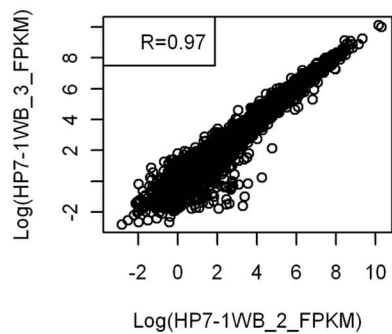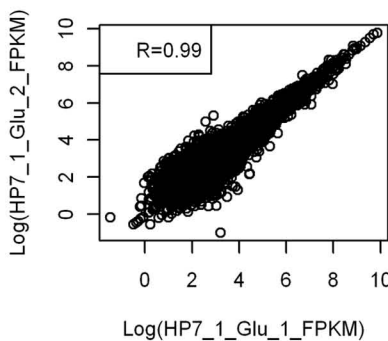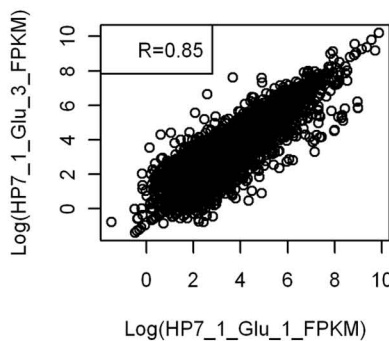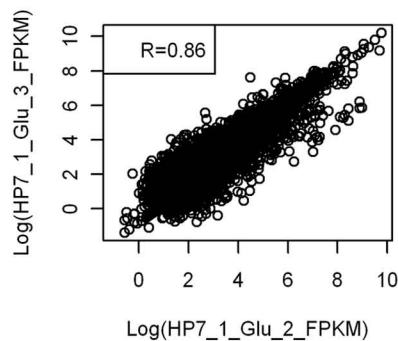

Supplement: Supplementary file 3 — Additional file 3: Figure S2. Pearson’s correlation analysis of the transcriptomes of P. oxalicum HP7-1 in the presence of glucose (Glu), wheat bran (WB), or wheat bran and Avicel (WA) as the carbon source. RNA for sequencing was extracted from cells sampled 72 h after inoculation. [file 13068_2017_966_MOESM3_ESM.pdf]

A

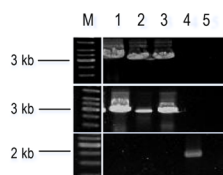

B

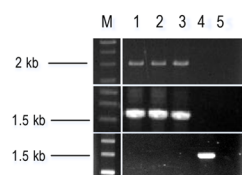

C

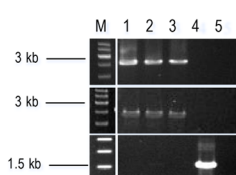

D

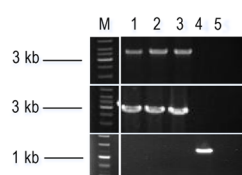

E

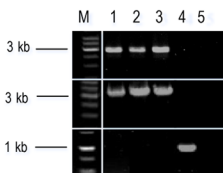

F

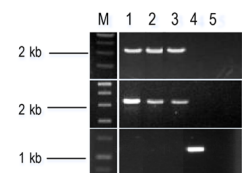

G

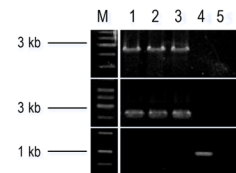

H

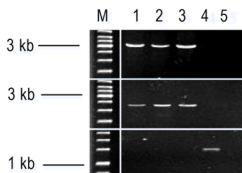

I

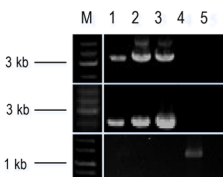

J

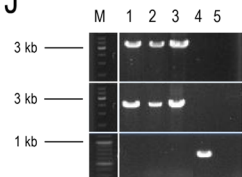

K

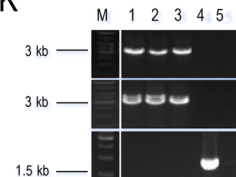

L

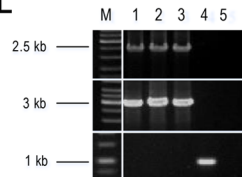

M

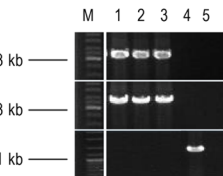

N

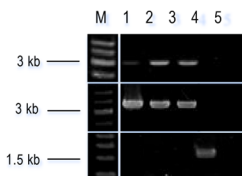

O

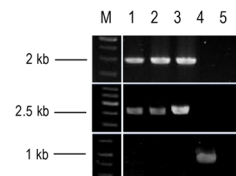

P

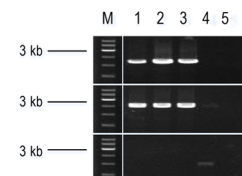

Q

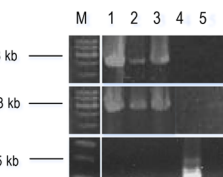

R

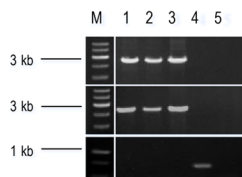

S

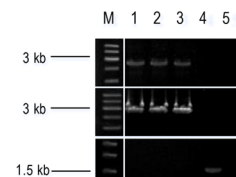

T

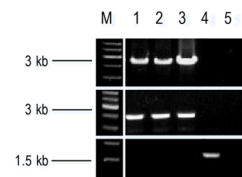

U

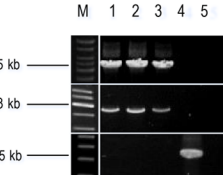

V

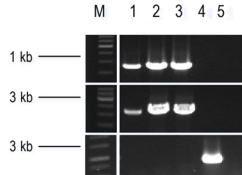

W

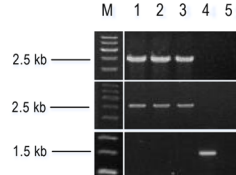

X

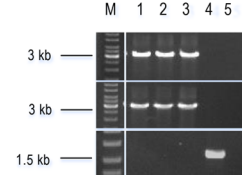

Y

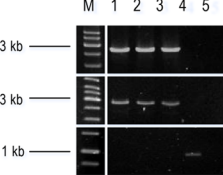

Z

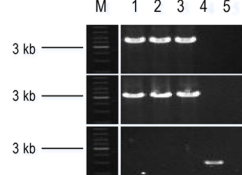

AA

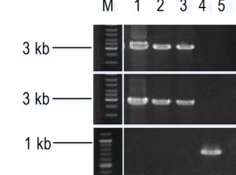

AB

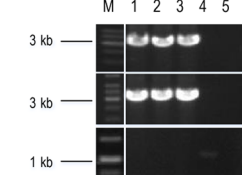

AC

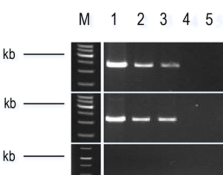

AD

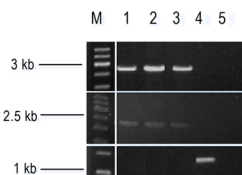

AE

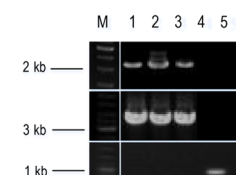

AF

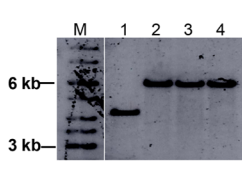

AG

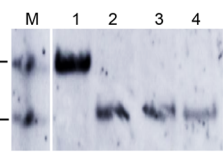

AH

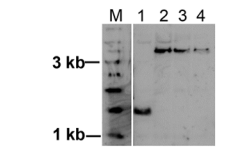

Supplement: Supplementary file 6 — Additional file 6: Figure S3. Confirmation analysis of the deletion mutants of 31 candidate genes derived from the parental strain ΔPoxKu70. (A–AE) PCR analysis of: (A) ΔPOX00864; (B) ΔPoxClrC; (C) ΔPOX01167/PoxCxrA; (D) ΔPOX01183; (E) ΔPOX01184; (F) ΔPOX02261; (G) ΔPOX02682; (H) ΔPOX02944; (I), ΔPOX03888; (J) ΔPOX03910; (K) ΔPOX04193; (L) ΔPOX04420/PoxCxrB; (M) ΔPOX04590; (N) ΔPOX04676; (O) ΔPOX04772; (P) ΔPOX04860; (Q) ΔPOX05374; (R) ΔPOX05436; (S), ΔPOX05726; (T) ΔPOX06377; (U) ΔPOX06396; (V) ΔPOX06425; (W) ΔPoxBrlA; (X) ΔPOX06759; (Y) ΔPoxFlbD; (Z) ΔPOX07934; (AA) ΔPOX08415/PoxNsdD; (AB) ΔPOX08702; (AC) ΔPOX08910; (AD) ΔPOX09356; and (AE) ΔPOX09460. M, 1-kb DNA marker; lanes 1–3, three transformants constructed for each candidate gene; lane 4, ΔPoxKu70; lane 5, ddH2O. (AF–AH) Southern hybridization analysis: (AF) ΔPOX01167/PoxCxrA; M, 1-kb DNA marker; lane 1, ΔPoxKu70; lane 2, ΔPOX01167/PoxCxrA-6; lane 3, ΔPOX01167/PoxCxrA-9; lane 4, ΔPOX01167/PoxCxrA-11. (AG) ΔPOX04420; M, 1-kb DNA marker; 1, ΔPoxKu70; 2, ΔPOX04420/PoxCxrB-2; 3, ΔPOX04420/PoxCxrB-6; 4, ΔPOX04420/PoxCxrB-8. (AH) ΔPOX08415; M, 1-kb DNA marker; lane 1, ΔPoxKu70; lane 2, ΔPOX08415/PoxNsdD-1; lane 3, ΔPOX08415/PoxNsdD-5; lane 4, ΔPOX08415/PoxNsdD-9. [file 13068_2017_966_MOESM6_ESM.pdf]

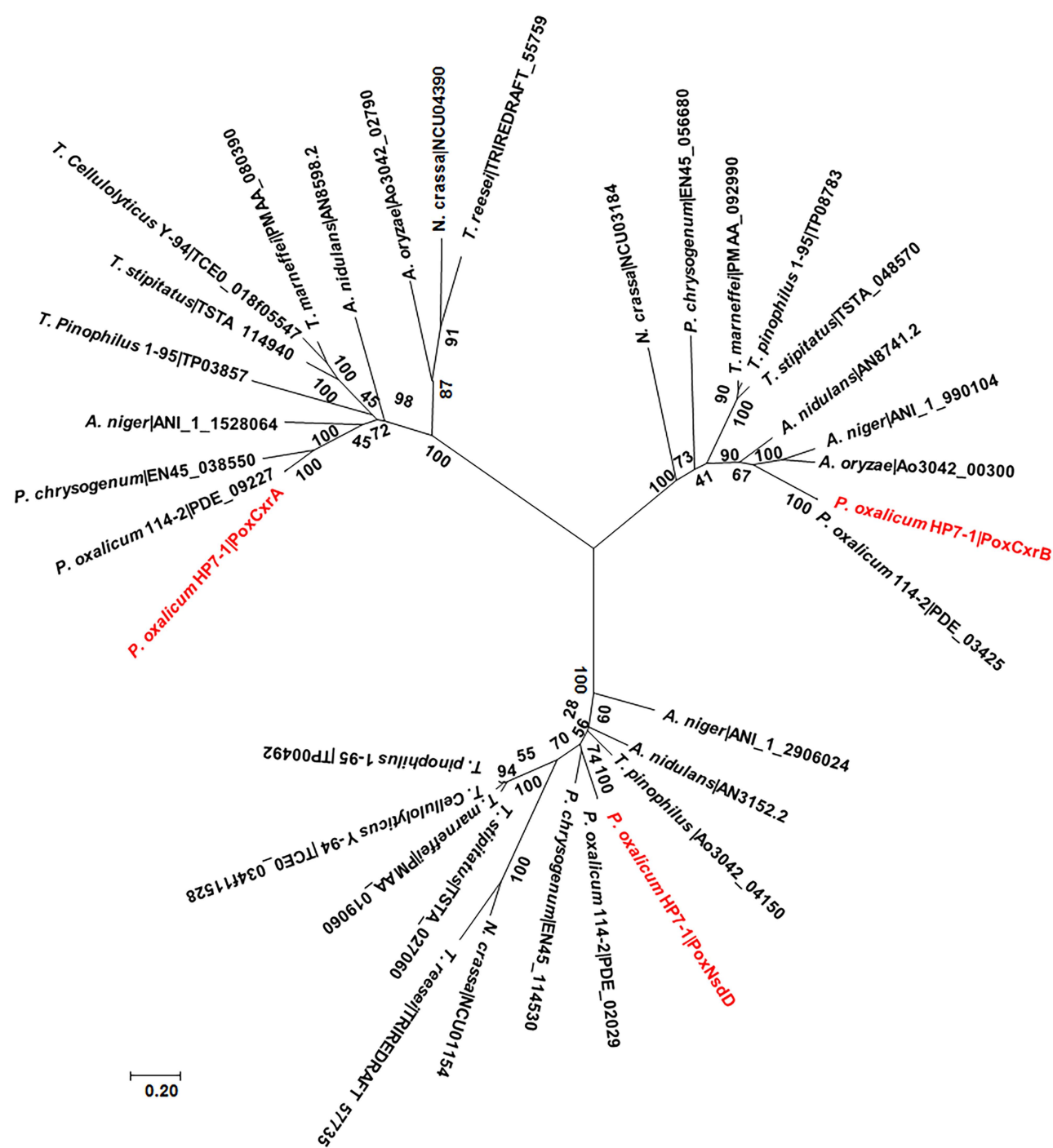

Supplement: Supplementary file 8 — Additional file 8: Figure S4. Unrooted phylogenetic tree of PoxCxrA, PoxCxrB, and PoxNsdD and their putative homologues. The dendrogram was constructed with the MEGA 7 software using the neighbor-joining method and a Poisson model. Bootstrap values shown at the nodes were derived with 1000 replicates, and the branch lengths, which correspond to the divergence of the sequences, are indicated by the scale bar. [file 13068_2017_966_MOESM8_ESM.pdf]

# PoxCxrA

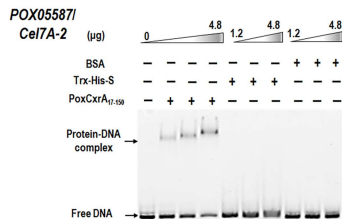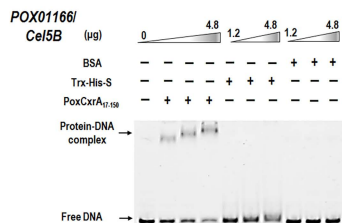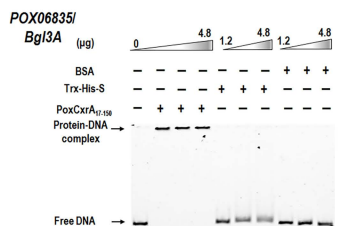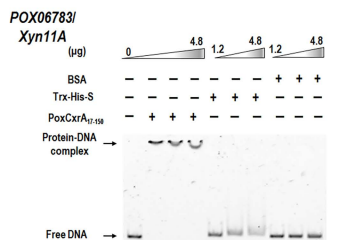

# PoxCxrB

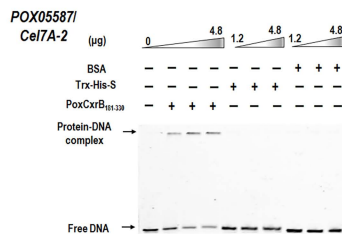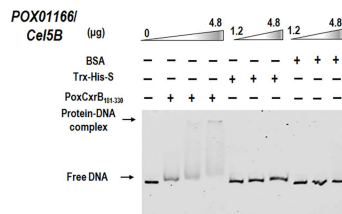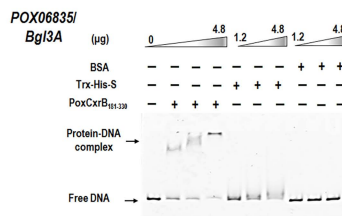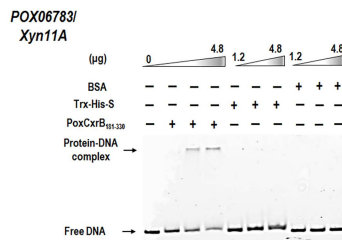

# PoxNsdD

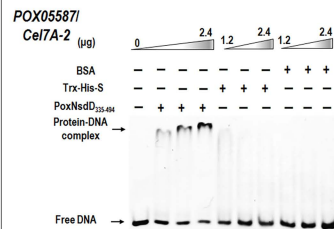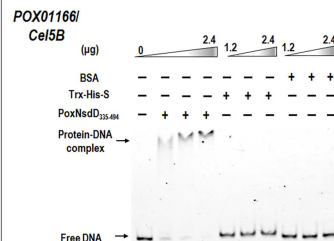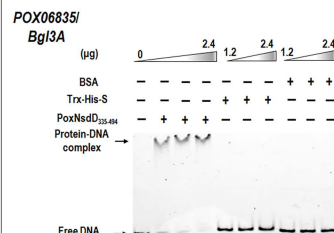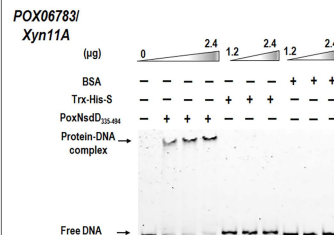

Supplement: Supplementary file 10 — Additional file 10: Figure S6. Electrophoretic mobility shift assay showing the interaction between the DNA-binding domains of the regulators and the promoter sequences of cellulase and xylanase genes. The experiments were performed without competitive probes. [file 13068_2017_966_MOESM10_ESM.pdf]

## PoxCxrA

### PoxCxrB

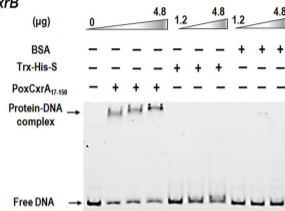

### PoxNsdD

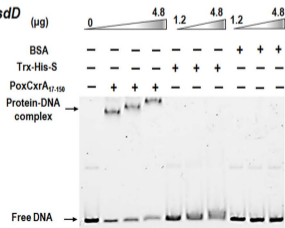

## PoxCxrB

### PoxCxrA

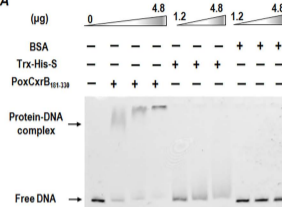

### PoxNsdD

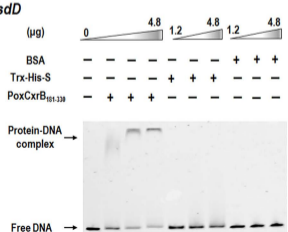

## PoxNsdD

### PoxCxrA

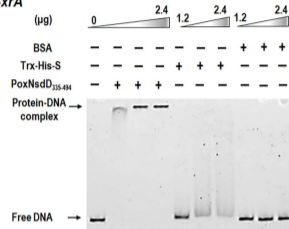

### PoxCxrB

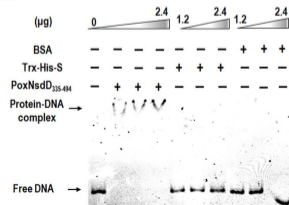

Supplement: Supplementary file 11 — Additional file 11: Figure S7. Electrophoretic mobility shift assay showing the interaction between the DNA-binding domains of the regulators and the promoter sequences of the newly identified regulatory genes. The experiments were performed without competitive probes. [file 13068_2017_966_MOESM11_ESM.pdf]
