# Supplementary material for: Transcriptomic profiling and genetic analyses reveal novel key regulators of cellulase and xylanase gene expression in Penicillium oxalicum
Source: Biotechnol Biofuels. 2017 Nov 22;10:279. doi: 10.1186/s13068-017-0966-y (PMC5700522; doi:10.1186/s13068-017-0966-y)
Supplement: Supplementary file 2 — Additional file 2: Table S1. Summary of RNA-sequencing reads obtained for P. oxalicum strain HP7-1 and its derived mutants. [file 13068_2017_966_MOESM2_ESM.pdf]

Additional file 2: Table S1. Summary of RNA-sequencing reads obtained for *P. oxalicum* strain HP7-1.

Samples	Clean Reads	Number of nucleotides (bp)	Quality Paired Reads	Overall read alignment rate (%)	Concordant pair alignment rate (%)	Number of expressed genes
HP7-1_Glu-1	26753840	2407845600	26207612	97.96	81.45	8055
HP7-1_Glu-2	26745472	2407092480	26120036	97.66	80.12	8063
HP7-1_Glu-3	27340898	2460680820	26756774	97.86	81.21	8243
HP7-1_WB-1	26645940	2398134600	24982716	93.76	80.36	9014
HP7-1_WB-2	26738590	2406473100	24959552	93.35	79.54	8867
HP7-1_WB-3	23395830	2339583000	20749677	88.69	65.9	8794
HP7-1_WA-1	26812882	2413159380	25081718	93.54	79.96	8947
HP7-1_WA-2	26758082	2408227380	24884526	93.00	79.4	8941
HP7-1_WA-3	27269148	2454223320	25637859	94.02	80.23	8671
