# Supplementary material for: Transcriptomic profiling and genetic analyses reveal novel key regulators of cellulase and xylanase gene expression in Penicillium oxalicum
Source: Biotechnol Biofuels. 2017 Nov 22;10:279. doi: 10.1186/s13068-017-0966-y (PMC5700522; doi:10.1186/s13068-017-0966-y)
Supplement: Supplementary file 4 — Additional file 4: Table S2. List of 108 genes differentially coexpressed on all carbon sources tested, including glucose (Glu), wheat bran (WB), and wheat bran and Avicel (WA). [file 13068_2017_966_MOESM4_ESM.pdf]

Additional file 4: Table S2. A list of 108 genes differentially co-expressed on all carbon sources tested, including glucose (Glu), wheat bran (WB), and wheat bran and Avicel (WA).

Gene_ID	CAZy_ class	Definition	Glu_1-FPKM	Glu_2-FPKM	Glu_3-FPKM	WB_1-FPKM	WB_2-FPKM	WB_3-FPKM	WA_1-FPKM	WA_2-FPKM	WA_3-FPKM
POX00001	NA	Hypothetical protein	78.42	41.28	37.97	328.98	277.64	351.25	131.55	173.27	136.04
POX00005	NA	Major facilitator superfamily	19.38	12.96	28.83	84.11	60.8	84.89	106.35	102.8	294.7
POX01798	NA	Hypothetical protein	81.65	72.58	92.7	230.38	160.34	191.59	425.41	411.57	993.11
POX01700	NA	Major facilitator superfamily	8.16	16.5	14.68	101.86	43.59	92.88	98.99	86.48	547.31
POX08089	NA	Major facilitator superfamily	3.05	8.41	6.23	41.87	42.73	45.41	82.36	101.44	418.5
POX01166	CBM1; GH5	Endo-β-1,4-glucanase	66.19	52.46	33.66	2379.01	2424.11	1866.97	10607.84	10426.03	1370.56
POX05571	CBM1; GH7	Endo-β-1,4-glucanase	17.69	12.65	9.65	537.11	554.18	479.66	2375.21	2156.14	318.91
POX01937	CBM1; GH5	β-1, 4-mannanase	25.52	29.33	12.19	425.06	431.08	374.72	1352.41	1412.14	295.09
POX05570	CBM1; GH45	Endo-beta-1, 4-glucanase Cel45A	10.88	20.02	22.4	1537.45	1186.3	1296.63	4294.82	5131.18	651.77
POX06079	GH1	intracellular β-glucosidase	19.4	24.97	28.58	645.79	783.76	566.74	2975.25	2439.68	511.49
POX09801	GH43	putative exo-β-1,3-galactanase	0.001	0.001	0.001	17.31	39.83	18.02	142.17	149.67	2.12
POX01896	CBM1; CBM46; GH5	Endo-beta-1, 4-glucanase Cel5C	14.17	13.03	12.16	371.04	559.86	397.88	1968.67	1928.04	128.3
POX05587	CBM1; GH7	Cellobiohydrolase CBHI/Cel7A-2	24.34	22.38	17.06	816.92	1209.68	598.45	9085.48	7067.58	224.8

POX06051	NA	Cellodextrin transporter Cdt-C	40.71	28.07	50.62	2266.73	2296.17	2116.07	5542.36	5279.5	2982.92
POX03430	GH11	Putative endo-β-1,4-xylanase	4.65	34.24	9.61	145.36	213.02	141.63	702.89	408.37	112.17
POX01704	NA	Hypothetical protein	45.87	22.71	15.97	129.15	100.38	118.93	388.69	393.35	58.72
POX02710	GH71	Putative α-1,3-glucanase	67.16	58.41	36.38	226.82	183.67	205.15	569.97	647.37	131.71
POX04786	CBM1; GH6	Cellobiohydrolase Cel6A	32.35	20.45	8.28	318.28	532.64	312.14	8903	7603.66	215.73
POX06147	CBM1; GH5	Endo-β-1,4-glucanase Cel5A	9.18	10.55	4.7	53.71	89.15	57.57	1014.85	836.35	39.33
POX08874	NA	Major facilitator superfamily	13.91	7.99	14.93	65.01	52.59	60.61	252.74	231.01	49.95
POX08485	CBM1	Swollenin	45.01	18.97	5.37	128.29	167.76	118.36	4164.55	4247.72	272.68
POX09387	GH18	Putative chitinase	112.22	108.49	25.99	462.08	422.94	512.05	2090.39	3211.4	708.11
POX07646	NA	Hypothetical protein	84.27	88.59	112.52	236.47	261.73	239.53	552.5	505.45	394.91
POX03641	GH3	Putative beta-glucosidase	40.65	28.28	35.86	162.74	197.16	148.68	621.74	559.99	243.52
POX06983	GH12	Xyloglucan-specific endo-β-D-1,4-glucanase	30.87	31.94	28.62	425.41	208.3	406.65	1622.67	2100.66	1375.65
POX07485	NA	Hypothetical protein	40.11	36.88	34.67	117.88	95.22	103.66	264.92	267.54	166.82
POX05915	NA	Cellodextrin transporter Cdt-D	92.36	91.44	93.7	524.11	577.57	618.22	1360.06	1225.68	1569.6
POX08652	NA	Hypothetical protein	15.63	11.97	16.02	49.37	52.45	45.5	102.91	116.15	113.32
POX05897	NA	Major facilitator superfamily	47.77	21.96	15.78	149.15	90.54	154.05	319.89	387.86	463.26
POX06607	NA	Hypothetical protein	6	5.52	11.46	29.19	67.39	31.7	182.29	127.15	13.21

POX02739	CE16	Putative carbohydrate acetylesterase	19.57	0.001	0.001	160.7	278.66	197.04	645.78	767.1	123.67
POX08897	CBM1; AA9	Putative cellulose monooxygenase	0.001	7.37	3.29	336.33	444.87	336.48	1323.74	1420.1	28.43
POX00860	NA	Hypothetical protein	22.21	34.04	0.001	1353.48	1719.19	1645.12	32.25	71.04	172.92
POX01912	NA	Hypothetical protein	14.03	4.3	0.001	619.07	707.75	786.28	187.79	169.38	0.84
POX01929	NA	Hypothetical protein	281.49	78.37	10.55	298.13	292.26	293.1	1709.04	1260.3	125.11
POX02308	AA9	Cellulose monooxygenase Cel61A	77.84	68.02	1.6	3543.15	4923.94	3592.43	14286.76	14944.47	834.55
POX02740	GH5	Putative endo-beta-1,4-glucanase	5.89	2.71	0.001	94.28	104.57	85.19	276.87	427.31	39.73
POX03540	NA	Hypothetical protein	9.97	4.59	0.001	41.47	69.7	35.91	170.21	185.03	10.73
POX00053	GH92	Putative α-mannosidase	483.66	317.87	90.58	14.56	29.31	14.85	136.75	125.76	12.4
POX02423	GH64	Putative β-1,3-glucanase	1278.98	1109.36	26.96	6.89	14.43	5.57	78.72	64.86	6.46
POX00105	GH55	Putative exo-β-1,3-glucanase	1372.4	1207.86	72.39	85.7	168.81	86.69	473.26	363.65	17.96
POX08190	NA	Hypothetical protein	1587.78	1309.71	62.1	41.64	77.06	40.25	378.68	320.7	18.57
POX06595	NA	Hypothetical protein	364.44	298.82	1009.76	24.21	55.76	15.45	361.15	168.63	3.57
POX07269	NA	Hypothetical protein	669.93	999.19	1180.04	15.95	24.1	20.1	105.64	74.02	1.14
POX07270	NA	Hypothetical protein	896.17	1115.11	2202.9	60.23	69.75	55.31	266.51	270.14	1.57
POX01168	NA	Major facilitator superfamily	905.69	983.72	274.01	3.43	6.68	3.77	79.37	45.09	4.74
POX01184	NA	Hypothetical protein	450.78	412.39	263.16	7.54	9.35	6.64	66.62	117.01	15.1
POX02008	NA	Hypothetical protein	1318.55	1450.59	1967.52	112.67	162.72	85.05	593.78	480.16	387.36
POX08451	NA	Hypothetical protein	268.14	137.97	237.51	4.53	7.43	2.92	43.05	43.26	22.84
POX08450	NA	Hypothetical protein	437.5	271.32	451.16	21.75	46.57	21.81	79.82	78.97	70.01

POX08646	NA	Hypothetical protein	573.15	351.74	571.13	22.25	42.44	31.58	121.68	109.65	96.27
POX07584	NA	Hypothetical protein	1951.21	2515.14	2523.75	17.35	13.1	14.37	110.46	113.79	151.97
POX02412	CBM20; GH15	Putative glucoamylase	277.73	296.94	349.66	55.82	65.97	62.75	172	116.54	95.6
POX03889	GH31	Putative α-glucosidase	220.93	252.98	348.49	13.56	14.77	12.47	84.53	38.4	20.94
POX09109	NA	Hypothetical protein	119.07	267.17	198.92	4.53	7.55	7.54	60.27	60.44	20.1
POX02066	NA	Hypothetical protein	255.1	343.73	343.19	6.46	5.34	5.59	76.4	85.03	48.31
POX02068	NA	Hypothetical protein	524.4	483.73	469.35	24.16	13.4	23.69	138.6	167.24	141.65
POX02069	NA	Hypothetical protein	400.5	489.21	415.79	0.14	0.14	0.83	25.9	33.02	17.68
POX08562	NA	Hypothetical protein	5074.44	3320.5	3050.87	283.04	278.95	379.27	1457.84	1419.65	2231.55
POX01891	NA	Hypothetical protein	363.54	373.98	282.28	76.47	51.3	81.67	97	88.6	255.76
POX05575	NA	Hypothetical protein	2922.89	1115.18	90.75	14.06	8.28	15.89	4634.59	5471.04	6494.63
POX08337	GH79	Putative β-glucuronidase	237.06	161.02	56.43	50.38	51.37	51.42	391.1	743.9	342.28
POX07573	GH3	Putative β-N-acetylhexosaminidase	155.89	136.06	132.25	63.48	32.09	51.27	299.8	335.78	364.3
POX09358	NA	Hypothetical protein	25.79	39.54	106.46	2.18	14.8	2.62	10.7	90.6	1195.35
POX01356	CBM20; GH15	Glucoamylase Amy15A	1281.59	1647.51	1235.33	569.98	893.23	629.79	563.99	227.51	113.01
POX07339	NA	Hypothetical protein	3326.49	3986.55	1389.5	49.25	124.38	69.54	20.87	18.53	8.73
POX05796	NA	Hypothetical protein	522.29	880.3	168.18	203.17	168.73	256.39	51.74	112.24	47.07
POX06151	NA	Hypothetical protein	281.27	358.86	130.3	81.75	61.56	82.31	8.63	15.19	3.45
POX09781	NA	Hypothetical protein	173.25	165.84	49.11	45.72	54.81	61.11	17.99	21.96	4.99
POX08906	NA	Hypothetical protein	16569.31	16351.17	9536.99	5869.69	6313.51	8779.25	2860.94	3267.6	4787.38
POX04781	NA	Hypothetical protein	5742.84	5486.58	6656.16	3445.14	1813.4	2962.85	162.22	250.22	838.97
POX05644	NA	Hypothetical protein	636.21	760.01	1455.95	435.68	228.07	356.01	137.69	136.73	174.08
POX07971	GH18	Putative chitinase	234.49	303.8	1089.44	242.63	134.83	206.86	68.31	85.79	86.03
POX04369	NA	Major facilitator superfamily	148.09	102.9	11.97	1405.05	2905.76	1397.29	1.79	1.53	0.61

POX00859	NA	Hypothetical protein	162.4	205.58	22.18	6007.85	9451.06	7250.16	129.61	284.88	1351.69
POX00863	NA	Hypothetical protein	30.24	31.79	7.13	920.49	1233.76	1071.41	38.09	55.99	142.4
POX02048	NA	Hypothetical protein	164.19	189.12	44.01	6789.47	4738.81	9152.08	281.21	477.5	2844.51
POX06862	NA	Hypothetical protein	67.64	62.19	41.95	565.86	450.91	530.28	92.67	97.48	434.05
POX00861	NA	Hypothetical protein	60.54	38.39	26.5	1603.47	2153.83	1731.95	127.94	158.52	217.87
POX00862	NA	Hypothetical protein	65.97	58.64	54.05	963.11	1394.71	1291.31	137.52	147	331.29
POX01691	NA	Hypothetical protein	43.56	28.03	33.9	325.59	297.32	417.81	59.63	108.54	149.55
POX01381	AA11	Chitin binding domain-containing protein	56.12	77.39	75.47	1784.05	1357.42	1388.22	252.11	333.13	839.86
POX07835	NA	Hypothetical protein	12.01	13.81	9.82	656.65	582.85	622.54	92.97	113.73	321.27
POX08030	NA	Hypothetical protein	168.45	154.92	166.72	888.6	825.61	1142.98	378.12	448.87	682.47
POX09026	NA	Major facilitator superfamily	22	51.48	31.92	564.05	495.59	636.49	142.06	177.57	315.46
POX04564	NA	Major facilitator superfamily	112.05	123.96	156.96	887.62	590.13	813.27	251.08	272.08	550.39
POX03381	NA	Hypothetical protein	43.61	48.14	7.2	691.39	1166.16	964.42	161.09	227.42	408.29
POX08136	NA	Hypothetical protein	43.65	51.84	15.62	350.96	327.23	407.05	160.32	156.1	226.61
POX05818	NA	Hypothetical protein	17.45	10.21	3.89	1450.35	1322.77	1304.24	100.65	96.12	293.2
POX07428	NA	Hypothetical protein	128.68	51.61	54.9	1249.61	1133.13	1174.63	386.03	366.1	490.2
POX01694	NA	Hypothetical protein	288.65	594.28	344.55	3267.31	2636.68	3667.9	1031.66	1608.66	1322.59
POX06052	NA	Hypothetical protein	14.48	19.23	14.47	219.57	193.19	209.78	81.4	95.74	43.06
POX09025	NA	Hypothetical protein	6.65	16.29	5.44	308.73	340.76	331.1	98.1	115.22	46.29
POX09210	NA	Hypothetical protein	92.84	104.51	65.34	1369.77	1605.61	1411.13	307.59	464.22	313.58
POX08478	NA	Hypothetical protein	30.26	38.98	140.78	3046.59	2427.61	3482.6	70.35	143.18	1553.46
POX01875	NA	Hypothetical protein	29.35	17.99	12.07	356.45	581.43	388.79	347.76	292.26	18.67
POX03738	GH114	putative endo-α-1,4-polygalactosaminidase	52.4	48.17	40.24	1653.37	1611.51	1819.25	771.37	726.44	55.76
POX09101	NA	Hypothetical protein	101.56	87.63	58.08	1054.04	1397.68	1259.43	452.04	802.62	128.36
POX08783	NA	Major facilitator	15.74	17.68	4.29	456.64	355.08	497.96	304.43	310.15	42.6

		superfamily								
POX03468	NA	Hypothetical protein	52.22	40.51	34.02	711.23	706.99	702.6	174.46	154.4	37.42
POX05578	NA	Hypothetical protein	118.68	94.25	8.85	20985.93	30062.07	21869.22	451.54	82.16	17.11
POX01886	NA	Hypothetical protein	4.45	0.001	14.69	387.9	267.55	375.46	175.33	179.53	12.35
POX09561	NA	Hypothetical protein	17.63	0.001	11.1	417.24	381.98	491.83	100.72	121.42	72.39
POX02591	CE16	putative carbohydrate acetylesterase	5.65	0.001	2.32	36.35	49.45	41.3	131.79	124.55	33.06
POX04137	CBM1; GH5	Putative endo-β-1,4-glucanase	19.78	18.19	74.48	466.07	276.01	365.82	175.37	194.99	187.87
POX07532	NA	Hypothetical protein	0.001	0.001	77.12	495.25	508.66	688.34	18.85	73.15	823.28
POX09814	NA	Hypothetical protein	0.001	0.001	7.49	294.03	232.74	264.53	66.76	102.95	109.6
POX07029	NA	Hypothetical protein	49.53	15.2	100.29	345.37	320.74	394.62	155.17	154.56	139.9

Note: CAZy, Carbohydrate-Active Enzymes; GH, Glycoside hydrolase; CE, Carbohydrate esterase; AA, Auxiliary activities; CWDE, Plant cell wall degrading enzyme; WA, wheat bran and Avicel; WB, wheat bran, Glu, Glucose, FPKM, fragments per kilobase of exon per million fragments mapped.
