# Supplementary material for: Transcriptomic profiling and genetic analyses reveal novel key regulators of cellulase and xylanase gene expression in Penicillium oxalicum
Source: Biotechnol Biofuels. 2017 Nov 22;10:279. doi: 10.1186/s13068-017-0966-y (PMC5700522; doi:10.1186/s13068-017-0966-y)
Supplement: Supplementary file 5 — Additional file 5: Table S3. Forty candidate transcription factors regulating the expression of cellulase and xylanase genes in P. oxalicum detected with a transcriptomic profiling analysis. [file 13068_2017_966_MOESM5_ESM.pdf]

Additional file 5: Table S3. Forty candidate transcription factors regulating the expression of cellulase and xylanase genes in *P. oxalicum* screened by differential transcriptomic profiling analysis.

Name	InterPro domain	Domain description	Known TFs	Log2(HP7-1_WB/ HP7-1_Glu)	Log2(HP7-1_WA/ HP7-1_Glu)	Log2(HP7-1_WA/ HP7-1_WB)
POX00864	IPR001138	Zinc finger, Zn2Cys6 type	NA	2.40	NT	-2.15
POX00936	IPR001138	Zinc finger, Zn2Cys6 type	NA	-1.65	NT	NT
POX00972	IPR004827	Basic-leucine zipper (bZIP) transcription factor	ClrC	2.56	1.77	NT
POX01167	IPR001138 IPR007219	Zinc finger, Zn2Cys6 type; Fungal_Trans	NA	-1.50	NT	NT
POX01183	IPR001138	Zinc finger, Zn2Cys6 type	NA	-4.55	-2.00	NT
POX01184	IPR011991	Winged helix repressor DNA-binding domain	NA	-5.58	-2.50	3.08
POX01907	IPR009057	Homeodomain-like	NA	-1.64	NT	NT
POX01960	IPR001138	Zinc finger, Zn2Cys6 type	ClrB	NT	1.62	NT
POX02261	IPR001138	Zinc finger, Zn2Cys6 type	NA	5.26	NT	-5.69
POX02338	IPR004827	Basic-leucine zipper (bZIP) transcription factor	NA	4.43	NT	NT
POX02682	IPR009057	Homeodomain-like	NA	1.84	1.65	NT
POX02768	IPR007087	Zinc finger, C2H2 type	PacC	3.15	NT	-2.28
POX02944	IPR001138	Zinc finger, Zn2Cys6 type	NA	-1.87	NT	NT
POX03120	IPR009071	High mobility group box (HMG)	NA	NT	1.53	NT
POX03888	IPR001138	Zinc finger, Zn2Cys6 type	NA	1.59	NT	NT
POX03910	IPR009057	Homeodomain-like	NA	-1.80	NT	NT
POX04193	IPR007219	Fungal_Trans	NA	NT	NT	-1.78
POX04420	IPR007087	Zinc finger, C2H2 type	NA	1.52	NT	NT
POX04590	IPR001138	Zinc finger, Zn2Cys6 type	NA	NT	-1.86	NT
POX04676	IPR001138 IPR007219	Zinc finger, Zn2Cys6 type; Fungal_Trans	NA	1.87	NT	-1.94
POX04769	IPR007087	Zinc finger, C2H2 type	NA	-2.56	NT	NT
POX04772	IPR009071	High mobility group box (HMG)	NA	-1.74	NT	NT
POX04860	IPR009057	Homeodomain-like	PDE_07199	-1.98	NT	NT

POX05374	IPR004827	Basic-leucine zipper (bZIP) transcription factor	NA	1.69	NT	NT
POX05436	IPR010982	Lambda repressor-like, DNA-binding domain	NA	1.61	NT	NT
POX05726	IPR007087	Zinc finger, C2H2 type	NA	-4.85	-4.66	NT
POX06377	IPR007396	Transcriptional regulator PAI 2-type	NA	2.20	NT	NT
POX06396	IPR007087	Zinc finger, C2H2 type	NA	-3.98	-4.50	NT
POX06425	IPR001138<br>IPR007219	Zinc finger, Zn2Cys6 type; Fungal_Trans	NA	NT	NT	-1.74
POX06534	IPR007087	Zinc finger, C2H2 type	BrlA	-5.58	-5.56	NT
POX06759	IPR001138	Zinc finger, Zn2Cys6 type	NA	-3.58	-3.14	NT
POX07099	IPR009057	Homeodomain-like	FlbD	-4.36	-3.73	NT
POX07934	IPR000679	Zinc finger, GATA type	NA	-1.77	-1.92	NT
POX08415	IPR000679	Zinc finger, GATA type	NA	-1.60	NT	NT
POX08522	IPR011991	Winged helix repressor DNA-binding domain	POX08<br>522	-2.25	NT	NT
POX08702	IPR011991	Winged helix repressor DNA-binding domain	NA	-2.73	NT	1.77
POX08910	IPR009057<br>IPR007526	Homeodomain-like; SWIRM domain	NA	-2.40	-2.06	NT
POX09312	IPR001138	Zinc finger, Zn2Cys6 type	NA	1.99	NT	NT
POX09356	IPR001138	Zinc finger, Zn2Cys6 type	NA	2.67	2.07	NT
POX09460	IPR002100	Transcriptional factor, MADS-box	NA	6.25	NT	-2.76

TF, Transcription factor; IPR, InterPro database (http://www.ebi.ac.uk/interpro/scan.html); Glu, Glucose; WA, wheat bran and Avicel; WB, wheat bran; NT, not tested; NA, not annotated.
