# Supplementary material for: Transcriptomic profiling and genetic analyses reveal novel key regulators of cellulase and xylanase gene expression in Penicillium oxalicum
Source: Biotechnol Biofuels. 2017 Nov 22;10:279. doi: 10.1186/s13068-017-0966-y (PMC5700522; doi:10.1186/s13068-017-0966-y)
Supplement: Supplementary file 7 — Additional file 7: Table S4. Primers used in this study. [file 13068_2017_966_MOESM7_ESM.pdf]

Additional file 7: Table S4. Primers used in this study.

Primer name	Sequence（5'-3'）
**Primers used for the construction of deletion mutants.**	
POX00864-left-F	AGAACAGAACGCTACACCCTCA
POX00864-left-R	GGTAATCCTTCTTTCTAGATGTCACCCACACACTCTTGC
POX00864-right-F	AATATCATCTTCTGTCGACAGATAGCTTTGACGGTCGCA
POX00864-right-F	CACGCCTCAGCCCAGAATC
POX00864-nest-F	CTGCGAGGGATTGGGTGAG
POX00864-nest-F	CGAGCGAAACCGTAGTGCG
POX00972-left-F	ATGGGTGAAGAGGGTTTAGGG
POX00972-left-R	TTTAGAGGTAATCCTTCTTTCTAGATACGCTTTTCAGTTGTA TATCCAGA
POX00972-right-F	TCCTTCAATATCATCTTCTGTCGACGTCCAGACTCCTTGCT CTTATGG
POX00972-right-R	AGTGCCAAGGTCGCAGTTCT
POX00972-nest-F	ATTCAAACACACGACATCATCTCTC
POX00972-nest-R	ATTCAAACACACGACATCATCTCTC
POX01167-left-F	GATCTGGTAGCCAATCCATTTC
POX01167-left-R	TTTAGAGGTAATCCTTCTTTCTAGAGGCCGACGACTTGAGA AACTAT
POX01167-right-F	TCCTTCAATATCATCTTCTGTCGACGGCAGCCCGCGGAGT G
POX01167-right-R	GACACGAAAAGGGGGGAAAAA
POX01167-nest-F	CAATGATGGCGAGTGGAGT
POX01167-nest-R	TCTGACTAAGTATGTCCCTTTGATT
POX01183-left-F	AGTCTTTGCGTTCTTGTTGTG
POX01183-left-R	GGTAATCCTTCTTTCTAGAAGTATTGACGGACTAAAGACAT
POX01183-right-F	CAATATCATCTTCTGTCGACTCATCCCACTGCACCAC
POX01183-right-R	TTGCCACAGTCAAGTCCTATCA
POX01183-nest-F	TTCACATGGCGTCAGGGTC
POX01183-nest-F	TGGAGTCGGAAAGGGTTG
POX01184-nest-F	GTTGGGCGGTACTATTCG
POX01184-left-R	GGTAATCCTTCTTTCTAGAGTTATGGGAGGGTAGATAA
POX01184-right-F	CAATATCATCTTCTGTCGACGAACTTTGAAGTATAGCG
POX01184-right-R	ATGGGATTTGTTTGCTCT
POX01184-nest-F	TCCGTTTGCTCACTTCTAC
POX01184-nest-R	GTTGGTGGCTCACGATAC
POX02261-left-F	TGGGATTTATTCACCCGGTTCATT
POX02261-left-R	TTTAGAGGTAATCCTTCTTTCTAGACACGACTTCACATTCGT TTGCCTCT
POX02261-right-F	TCCTTCAATATCATCTTCTGTCGACAAGCAAAATCTCATTAG ATTGTTGA
POX02261-right-R	GATAGCCCATGATAGTTTGTTGC
POX02261-nest-F	CACCACTCGTATGGCAAGTCGTT
POX02261-nest-R	GGCAACCTCAGACTTCCTTCATAAT
POX02682-left-F	CTTCTAATGCGTCATCTGAAAACTC
POX02682-left-R	TTTAGAGGTAATCCTTCTTTCTAGAACATCGACCTTGCCGG G
POX02682-right-F	TCCTTCAATATCATCTTCTGTCGACAATCAGCGCCATTTTAT TATCTTCA

POX02682-right-R	GGAAACGCCTTCTCGTGTGC
POX02682-nest-F	CAGGAAATGACTGCTGAGTTGGAAA
POX02682-nest-R	GAAGACTGGCTTGGACGGCA
POX02944-left-F	CTGAGCGAAAGCACCCTATG
POX02944-left-R	GGTAATCCTTCTTTCTAGACGAAGTCGAACCGGCAGCAAG
POX02944-right-F	CAATATCATCTTCTGTCGACCCTCTCTCCTCCCTCTCCCTCTC
POX02944-right-R	CAACCAATCGGAGCGAGTAAAGT
POX02944-nest-F	CCAGGTCGTAGTCATCCGTAAC
POX02944-nest-R	CTTGCGTTGGCTTGGTCGTCGTA
POX03888-left-F	GAAAATAGGAAGAAACAAGAAGGG
POX03888-left-R	GGTAATCCTTCTTTCTAGATGCTACTTATCATGAAAATGGTCGT
POX03888-right-F	AATATCATCTTCTGTCGACGATGATTCAAGGAGGAAAACAA
POX03888-right-R	TCTCCCCTAACGCGACTCAC
POX03888-nest-F	CCATGTCCATGCCGTCCG
POX03888-nest-F	TCAAGCCCGCCATTCTCG
POX03910-left-F	TACGATGGATTACGACGGTTCA
POX03910-left-R	GGTAATCCTTCTTTCTAGAAGTCTTCGACGACGTTTGTGGCA
POX03910-right-F	CAATATCATCTTCTGTCGACGGGACGCCTCTCTTATCGACTGT
POX03910-right-R	TCTGAGCAGGGATGTCG
POX03910-nest-F	GGGTGGTCAACGTGGTAG
POX03910-nest-R	TGGATTATCTGGGATGAGTT
POX04139-left-F	AGTTCCCTGCCTATTCAAGC
POX04139-left-R	GGTAATCCTTCTTTCTAGACGACCAGCGAAGAAATAATCA
POX04139-right-F	AATATCATCTTCTGTCGACGAAATTGATATTTGCATGAGCG
POX04139-right-R	CCATCAAATAATCCGTCTCC
POX04139-nest-F	ACAGTCGCAATGAGGAAATG
POX04139-nest-F	GTAGCAGCAGTGTTCTCGTTC
POX04420-left-F	GCCAACATCAAAGTCCGATAACA
POX04420-left-R	GGTAATCCTTCTTTCTAGATGTCGGACTAGATGCTTCGT
POX04420-right-F	AATATCATCTTCTGTCGACGACAACAAAAAACCCCCTTCA
POX04420-right-R	CGATTCGTGCGTGATGTTGAG
POX04420-nest-F	ATACAAACCAAACGAGAAAGTGAA
POX04420-nest-R	CGTCCTCGTTCCACTTGCG
POX04590-left-F	GCATCCGAGGCATTAGC
POX04590-left-R	GGTAATCCTTCTTTCTAGATCGAAATGGATGATGCTC
POX04590-right-F	CAATATCATCTTCTGTCGACGAATCAGGGGTAAAATTACCCCGC
POX04590-right-F	AATGGGTAAAGTGGACTGACG
POX04590-nest-F	GGGGAATAGTGGAACAAATGG
POX04590-nest-R	GCCTTAGTGTCCTTAGCGTGATAG
POX04676-left-F	TGATCCAAGGGCAGATGACT
POX04676-left-R	GGTAATCCTTCTTTCTAGATGCCGCACGTGGTCGA
POX04676-right-F	AATATCATCTTCTGTCGACCTGCTTGATTCGGAGAAGGG
POX04676-right-R	CCTTCCGAGACGAGGGTGATA
POX04676-nest-F	ATAATAGGTGATTTTCTTACGGTCG
POX04676-nest-R	TCCCAGCCTTCCTCGGTATAAT

POX04772-left-F	TCGGAACCTCCTCCATCTG
POX04772-left-R	GGTAATCCTTCTTTCTAGATGATGAAGAACAAGTGAT
POX04772-right-F	AATATCATCTTCTGTCGACACGGCTCGGTATATTAGAT
POX04772-right-R	ATGAAGCGGTCTTGGACG
POX04772-nest-F	GGAACCTCCTCCATCTGAAT
POX04772-nest-F	ATGGATGACCAGCACCGT
POX04860-left-F	GCGATAGAAGCCGAGACCA
POX04860-left-R	GGTAATCCTTCTTTCTAGAGGCGAAGAAGAAGGAATGT
POX04860-right-F	CAATATCATCTTCTGTCGACCAGGGTCTCGACCAGTCATGGT CTC
POX04860-right-R	AGAGCCTGGGCACTACGAG
POX04860-nest-F	GAAGGGCAGTAGGAAGTGA
POX04860-nest-R	ACTCGGCTGCGTTGTGGTA
POX05374-left-F	GGGTGAACTTCTTCGCTCTA
POX05374-left-R	GGTAATCCTTCTTTCTAGAGGTTGGATCCTTTCGTCGGGCC
POX05374-right-F	CAATATCATCTTCTGTCGACATTACACCTGTCGACCTGATCTG
POX05374-right-R	TGGCACTCCCATCGGTCT
POX05374-nest-F	GGGTGAACTTCTTCGCTCTA
POX05374-nest-R	ACCATTTGCCGCCTCTG
POX05436-left-F	GGCTTCACTTCGTTGTGTTCC
POX05436-left-R	TTTAGAGGTAATCCTTCTTTCTAGAGGTGGATAAGATTTTGATCAGGCT
POX05436-right-F	TCCTTCAATATCATCTTCTGTCGACGTCGTTTTCTGGAAAGCAATTTT
POX05436-right-R	GAAGCATAAAAACCTGACCGC
POX05436-nest-F	GCCGCTCTTGCTGGGTCT
POX05436-nest-R	CAAGTCAAATGAAGCCCAGAAA
POX05726-left-F	AACGGATTGAGGGAACGAGAG
POX05726-left-R	TTTAGAGGTAATCCTTCTTTCTAGAGATGTGGCTGAAGGGTGGC
POX05726-right-F	TCCTTCAATATCATCTTCTGTCGACGGGTGTGGATATCGAGCT GG
POX05726-right-R	ACGCAGGAAGGGACTCGC
POX05726-nest-F	TTGCTCGGGAGTTTTTCAGGTTC
POX05726-nest-R	GAGATGACGGATTTTGAGGACCC
POX06377-left-F	CCGACGAGGTCTCGCAGGG
POX06377-left-R	TTTAGAGGTAATCCTTCTTTCTAGAGGTCAGCAGGATGGAATCACTCAG
POX06377-right-F	TCCTTCAATATCATCTTCTGTCGACAGTGTATGACTTTTGAGGTCCTATT
POX06377-right-R	CCCGTACAGCTGGCATATC
POX06377-nest-F	ATTCTGTGCTGTGATTGGTGTGC
POX06377-nest-R	GGTCAGACCACCCCTGGATAATAT
POX06396-left-F	TCACCCATCGCTCTTCACTC
POX06396-left-R	TTTAGAGGTAATCCTTCTTTCTAGATATGGTGTCACGGATATGGGA
POX06396-right-F	TCCTTCAATATCATCTTCTGTCGACAGCTTCCTCTCCGTCGAGC
POX06396-right-R	TTCCTATTTTCACTCATTCTTCCAG

POX06396-nest-F	CGGTTATGGTGTCTCCTTTCTCT
POX06396-nest-R	CACGCCGTTGGACTTGACA
POX06425-left-F	CAACGCAACGGAATGGC
POX06425-left-R	GGTAATCCTTCTTTCTAGACTTGGCAGAGCAAAGACAGGCGCAT
POX06425-right-F	CAATATCATCTTCTGTCGACTGAATTCAGACTCGGATTCTC
POX06425-right-R	GAGACCCGAGCGAACATTA
POX06425-nest-F	ATCACTCCCTACCCAGCAAC
POX06425-nest-F	CCGAACAAGAAACCACCC
POX06534-left-F	GTGTGACGGGACATACAGTGG
POX06534-left-R	TTTAGAGGTAATCCTTCTTTCTAGAGTCGTCGAGTTCTTCAATTTTG
POX06534-right-F	TCCTTCAATATCATCTTCTGTCGACATGGACGGCGCTCTGTTCT
POX06534-right-R	GGGTAAGTCTCAATGCAGGGTT
POX06534-nest-F	ACTGGGAAAATAAGGACACCAAAAA
POX06534-nest-R	TGAGGCAGTGTTTGCTCTTGGT
POX06759-left-F	TCCTACGCTTGAACTTGAACGA
POX06759-left-R	GGTAATCCTTCTTTCTAGAGCAACGACTAATCAAATA
POX06759-right-F	AATATCATCTTCTGTCGACACGGGTGGTAATTTGAA
POX06759-right-R	ACACAAACAAACCACGAGTCCAC
POX06759-nest-F	AGCCTGGTTCTATGAGTCTCCGTC
POX06759-nest-R	TGGCGAACAGATGCGACAC
POX07099-left-F	AAGAGAAAAGGGTCAGAGACAGGAG
POX07099-left-R	TTTAGAGGTAATCCTTCTTTCTAGAGGTGGGGGGTGAGGGGT
POX07099-right-F	TCCTTCAATATCATCTTCTGTCGACTTGTCCAAGAATCCGACTGTC
POX07099-right-R	GGGTCAGCCGTCCTAAGAA
POX07099-nest-F	GATCTGCCTGAAAAGACGTCGCT
POX07099-nest-R	CGCAAACTGGACGGAGAGGGT
POX07934-left-F	AGCCCGCCCGCATTAGAT
POX07934-left-R	GGTAATCCTTCTTTCTAGAGACGCTATCAGGAGCAGGCGGCAG
POX07934-right-F	CAATATCATCTTCTGTCGACAAACTTATGATGTTTTCCCCTCCCC
POX07934-right-R	CTACCAGGTCCGCTCTTGCTTCTAT
POX07934-nest-F	ACATGCCCTGCGAAAGTC
POX07934-nest-R	ACCCTCCCAACGAGAAGT
POX08702-left-F	CGTCTCAAGGTTGGCAGGGT
POX08702-left-R	GGTAATCCTTCTTTCTAGAGCTGAGTACAGGTATGGTGA
POX08702-right-F	AATATCATCTTCTGTCGACATGTCCTTTCAATTCTGTAG
POX08702-right-R	AACTTGTAAATGGCGTGGATGG
POX08702-nest-F	ATGGTGCTGTAATAGGGTTAGAGAG
POX08702-nest-F	GGACATTCATCAACGACAAAGACTA
POX08415-left-F	AGTGCCGATGTCGTCCTGTTCTC
POX08415-left-R	GGTAATCCTTCTTTCTAGAATCGACGATCGGCGATTTGG
POX08415-right-F	CAATATCATCTTCTGTCGACTTTTGTCGATTTTGTTTCACTTTT
POX08415-right-R	CTCGGATAGACAAGAAATAAGC

POX08415-nest-F	CCGCCGTCTCATCCTCA
POX08415-nest-R	GACCCAAACATACTGCTTCCA
POX08910-left-F	AATCATCGTCCGCACCTTA
POX08910-left-R	GGTAATCCTTCTTTCTAGAGATGGAAGAGTAAGAAGGGGA GTGG
POX08910-right-F	CAATATCATCTTCTGTCGACATTTTGCCTCGCACTCACTAG
POX08910-right-R	CCACCTCCCACGGCTTGTA
POX08910-nest-F	CCTGCTTGTATGAATGACGG
POX08910-nest-R	ACTGGCTGGTTTGCGGGAT
POX09356-left-F	AAGACGCTTGGCACACGC
POX09356-left-R	TTTAGAGGTAATCCTTCTTTCTAGAGGTAAGATCGATATCGT GTACTTGT
POX09356-right-F	TCCTTCAATATCATCTTCTGTCGACATTGAAGATCAGGGGA GAGAGAGAG
POX09356-right-R	GGAACTACCTGGTACATCCGCAA
POX09356-nest-F	ATCAAATATCCGCATGTCCACCT
POX09356-nest-R	ACCTGGTACATCCGCAAGTCACT
POX09460-left-F	GCATACGATGTCACTTGGCG
POX09460-left-R	GGTAATCCTTCTTTCTAGACTTAGTCTTGCCTCGTTTTAG
POX09460-right-F	CAATATCATCTTCTGTCGACGGACAATTCAACAAGTCCTCG
POX09460-right-R	TTGCCAACCCACTCCTCTG
POX09460-nest-F	CCATGCCCCAGAAGTTAC
POX09460-nest-F	TTGATTGGTGCGTCGTAA
G418-F	TCTAGAAAGAAGGATTACCTCTAAA
G418-R	GTCGACAGAAGATGATATTGAAG
**Primers used for confirmation of deletion mutants.**	
POX00864-F	AACGAGCGTCACTACCAGAG
POX00864-F	CAGCACCCGTCAATCACTT
POX00972-F	TGGACCACGATGGCTGACC
POX00972-R	CGTCCATAGCAGCAGCGTC
POX01167-F	CCAAAAGGTATGGGGGGATG
POX01167-R	GGTAGCACAAATGGCGTCCC
POX01183-F	CACCTCGGGAAGCACTCA
POX01183-R	AAGCGGTCCACGATAGCC
POX01184-F	TTATCGGGGTCTCAGAAAGTC
POX01184-R	GGTTCACCAGCACCATCC
POX02261-F	CTCGCCGCTCCCATCAG
POX02261-R	TTTGAACAATCTCACCAAGCACTAC
POX02682-F	TTTCATTGGCAGATCCATACCG
POX02682-R	GTCTCCAGGCATCGATTCACG
POX02944-F	GAGACCAAGCCAGCGTAA
POX02944-R	CCGAACAGCCGAGTAAGA
POX03888-F	TTCGTGAGATGGCTCGTATT
POX03888-R	CCGTTGGATGTTATGTTGGA
POX03910-F	CCTCGGCGTCAAGTATCAC
POX03910-R	ATGGACAAGGAACGGCAC
POX04193-F	CGCTCCCCGAGGTATGTC
POX04193-R	TCCCAATCCCACCTCGGC
POX04420-F	TGCCCAAGTCATACAGTGAAACA
POX04420-R	CGGAGAATGCCTTGCCACA

POX04590-F	CTCATCCATCGGGACCATCACA
POX04590-R	GTCGCCTTCCAAACTCAGCATT
POX04676-F	ACAACGCCATCCGAGCCT
POX04676-R	CGCATCCTTGGTGTCCAT
POX04772-F	ACCGACAGCAAGCCTACC
POX04772-R	GGGACATCTTCGCCATACA
POX04860-F	CTGAGTCTGCCACGAGTTG
POX04860-R	GACCCGAGGAAGGATTGC
POX05374-F	CATCGGCATGGGATCAGC
POX05374-R	GAGGACGGGAGAACAGGA
POX05436-F	TCGATGTATGTCACTCCTTCCCCTC
POX05436-R	CCACTTTCCTCTCAACGTAATCACC
POX05726-F	TGTCCATTGTCATCCCTGCCT
POX05726-R	GTTCGCTTGGGACAAACATCAC
POX06377-F	ATGTACTCCCAATCCGTTCACG
POX06377-R	TCTTTTGAGAGTCCTTCTTCATTCC
POX06396-F	TTACAACCCGTCCTTTGACACCC
POX06396-R	TTCCACGGCGTCGTTGCTT
POX06425-F	ACGAGCGACAAACTAAGCC
POX06425-R	TAACTCACCTGCGACGACA
POX06534-F	ATGAGGACACAAGGTCAGCAAATCT
POX06534-R	CCCAGCCATCCACGCTAAGA
POX06759-F	AGTTCGCCACCTGCGTTCA
POX06759-R	TCCTCCTCCTCCTTCTTCGC
POX07099-F	ATGGCTCCCTCCCACCGT
POX07099-R	TGAGGAGATTGTTCAGGCCCA
POX07934-F	ATTCCAACAACCTACCCTTCC
POX07934-R	CCGCTCTTCTTCGTGCC
POX08415-F	GCGTGTTCTGCGTGTCA
POX08415-R	TTGCGAGTCAGTTTAGCG
POX08702-F	TTGACGCATACCAAGCAGA
POX08702-R	ATCAGTCAACGCTTCCTTT
POX08910-F	GGGTGGCAATGCTAAGGC
POX08910-R	CGGCAAGTGGCGGAAATAC
POX09356-F	GGGGTTCGGGAAGCAGG
POX09356-R	GTGACCCCCATATCTCCCTGA
POX09460-F	CTATGCCGTGGCTTCCTTTG
POX09460-F	TTCCCTACGCCCACAATCC
POXG418-F	GATAATAATGTCCTCGTTCCTGTCT
POXG418-R	ATGCTCCTCTTCTTTACTCTGATAG
**Primers used for construction of complementary strains**	
CxrA-L-F	ACATCCAAGAATCTACTGTTCCCTG
CxrA-R	TTAGAGGTAATCCTTCTTTCTAGAACGCCAGGAATGCTGCTTT
CxrA-R-F	GGATCTTACCGCTGTTGAGATCCAGCCTTCCATTGGCCTGCATT
CxrA-R-R	CACGATTGAATGAATGACCCTC
C-CxrA-F	AATCTATTTGGGTCCCGCTGC
C-CxrA-R	ACCTCAGTCCACGAATCACGGC
CxrB-F	GACCACTTACTCTGTAGGTGATTGC

CxrB-R	ATGATGCGGGTGTCGGT
PepA-L-F	AAGCTTGGTACCGAGCTCGGATCCATTACTTAAGATTACGGGCTG
PepA-L-R	AATTCCAGCACACTGGCGGCCGTTACTGGATCTCAACAGCGGTA
PepA-R-F	TTCTGCAGATATCCATCACACTGGCATGTAGATCACCAAGTTGCA
PepA-R-R	GGGCCCTCTAGATGCATGCTCGAGCCGAATAGAAGATAACCCACG
C-PepA-F	TTACTTAAGATTACGGGCTG
C-PepA-R	GTGCGAGGCGTGATAAGAAG
NsdD-F	CTTGCGTGACCATTTCTCC
NsdD-R	TTTCCCTCCTGATGATTGTA
Ble-F	TCTAGAAAGAAGGATTACCTC
Ble-R	CTGGATCTCAACAGCGGTA
CxrA-CDS-F	CCAAAAGGTATGGGGGGATG
CxrA-CDS-R	GGTAGCACAAATGGCGTCCC
CxrB-CDS-F	TGCCCAAGTCATACAGTGAAACA
CxrB-CDS-R	CGGAGAATGCCTTGCCACA
NsdD-CDS-F	GCGTGTTCTGCGTGTCA
NsdD-CDS-R	TTGCGAGTCAGTTTAGCG
PepA-CDS-F	ATGGTTGTCTTCAGCAAGGTTA
PepA-CDS-R	CTATGCCTGAGCAGCGAAA
**Primers used for probe amplification for Southern hybridization**	
PoxCxrA-probe-F	CTATTTGGGTCCCGCTGCCT
PoxCxrA-probe-R	GCCCTGTATTTCCACAATGCCT
PoxCxrB-probe-F	ATACAAACCAAACGAGAAAGTGAA
PoxCxrB-probe-R	CCACCCGCCGAGCATCAGT
PoxNsdD-probe-F	AAGATTCAAGCGAAAAGGACA
PoxNsdD-probe-R	CCAGATGGCGAGACAGGTAT
**Primers used for amplification of DNA sequence encoding DNA-binding domain in PoxCxrA, PoxCxrB and PoxNsdD.**	
PoxCxrA$_{17-150}$-F	GGAATTCCATATGGCATGCGTGCTGTGTCA
PoxCxrA$_{17-150}$-R	ATAAGAATGCGGCCGCATGGCGCAGATCATCGTT
PoxCrxB$_{181-330}$-F	ACGCGTCGACGTTCCACCTCTTCTCACTCCTCCTAC
PoxCrxB$_{181-330}$-R	CCGCTCGAGTTAAGAGACCATGGCCTGCAGAGCTGC
PoxNsdD$_{335-494}$-F	ACGCGTCGACGTGCGCATCAGACGCAACGCGCTGG
PoxNsdD$_{335-494}$-R	CCAGATGGCGAGACAGGTAT
**Primers used for amplification of probes for electrophoretic mobility shift assay Applied in PoxCxrA$_{17-150}$ binding**	
POX05587-P-F	TCCTTCCTCATCTCCTCCACC
POX05587-P-R	FAM-AGGGAGGAGAGGAGGAGAGC
POX01166-P-F	TTAGGAGTGCTATAAGATGGCGA
POX01166-P-R	FAM-CAACTTACAGACAAAGCCTCCG
POX06835-P-F	AGGTTCAAGTTGGCTTACCAG
POX06835-P-R	FAM-CAGATGAACGGACGCTTGT
POX06783-P-F	CCTCGGTCCGCATTTGGC
POX06783-P-R	FAM-TGTTTTATTCAAAGGAACGATG
PoxCxrB-P-F	CACCTTATCGGTCGTGACTAAATCT
PoxCxrB-P-R	FAM-GTGAAAGAGGTGCTTGTCGTGA

PoxNsdD-P-F	GTTATCTTACCAACCTTGTCATCGT
PoxNsdD-P-R	FAM-CGGCTTCCAAAGACCCCATA
**Applied in PoxCxrB$_{181\text{-}330}$ binding**	
POX05587-P-F	GAGAGATCCACTACCCGCAA
POX05587-P-R	FAM-AGGGAGGAGAGGAGGAGAGC
POX01166-P-F	TGGCAGACGCAGTCACGC
POX01166-P-R	FAM-CAACTTACAGACAAAGCCTCCG
POX06835-P-F	AGGTTCAAGTTGGCTTACCAG
POX06835-P-R	FAM-CAGATGAACGGACGCTTGT
POX06783-P-F	CCTCGGTCCGCATTTGGC
POX06783-P-R	FAM-TGTTTTATTCAAAGGAACGATG
PoxCxrA-P-F	TTCTAAAACCCGCCTGTCCC
PoxCxrA-P-R	FAM-CGGTCACATTTGCTCTTGCG
PoxNsdD-P-F	CCTCATCATCACCCATCGTCA
PoxNsdD-P-R	FAM-CGGCTTCCAAAGACCCCATA
**Applied in PoxNsdD$_{335\text{-}494}$ binding**	
POX05587-P-F	AGTGCCCTACGAGACTTCCC
POX05587-P-R	FAM-GGATTGGATCAAAGATCAAAAGAG
POX01166-P-F	TGGCAGACGCAGTCACGC
POX01166-P-R	FAM-CAACTTACAGACAAAGCCTCCG
POX06835-P-F	GGATTCAAACGGTAAAAATGGAG
POX06835-P-R	FAM-CAGATGAACGGACGCTTGT
POX06783-P-F	CCTCGGTCCGCATTTGGC
POX06783-P-R	FAM-TGTTTTATTCAAAGGAACGATG
PoxCxrA-P-F	TGCGTGATGAAGGCATTGTG
PoxCxrA-P-R	FAM-CGGTCACATTTGCTCTTGCG
PoxCxrB-P-F	ATCCACACAATGGGACTGCTC
PoxCxrB-P-R	FAM-GTGAAAGAGGTGCTTGTCGTGA
