# Supplementary material for: Transcriptomic profiling and genetic analyses reveal novel key regulators of cellulase and xylanase gene expression in Penicillium oxalicum
Source: Biotechnol Biofuels. 2017 Nov 22;10:279. doi: 10.1186/s13068-017-0966-y (PMC5700522; doi:10.1186/s13068-017-0966-y)
Supplement: Supplementary file 9 — Additional file 9: Figure S5. Confirmation analysis of the complementary strains. Targeted complementary genes including PoxCxrA, PoxCxrB and PoxNsdD were amplified using primer pairs CxrA-CDS-F/CxrA-CDS-R, CxrB-CDS-F/CxrB-CDS-R and NsdD-CDS-F/NsdD-CDS-R. Bleomycin resistance gene was amplified using primer pair Ble-F/Ble-R. M, 1-kb DNA marker; lane 1, complementary strain; lane 2, ΔPoxKu70; lane 3, corresponding deletion mutant strain. [file 13068_2017_966_MOESM9_ESM.pdf]

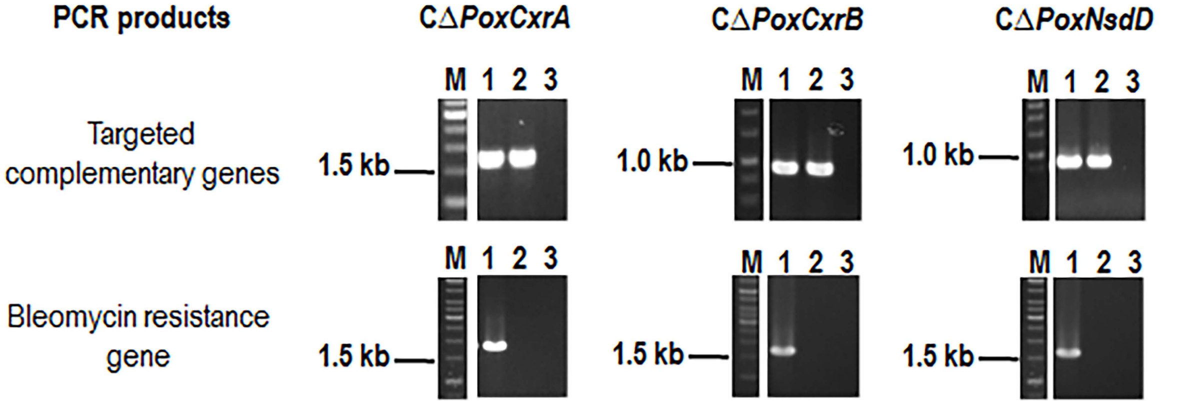
PCR products
CΔPoxCxrA
CΔPoxCxrB
CΔPoxNsdD
Targeted complementary genes
Bleomycin resistance gene
M 1 2 3
1.5 kb
1.0 kb
